# Fluorescent Magnetopolymersomes: A Theranostic Platform to Track Intracellular Delivery

**DOI:** 10.3390/ma10111303

**Published:** 2017-11-13

**Authors:** Oliver Bixner, Noga Gal, Christoph Zaba, Andrea Scheberl, Erik Reimhult

**Affiliations:** 1 Institute for Biologically Inspired Materials, Department of Nanobiotechnology, University of Natural Resources and Life Sciences, Muthgasse 11, 1190 Vienna, Austria; oliver.bixner@boku.ac.at (O.B.); noga.gal@boku.ac.at (N.G.); andrea.scheberl@boku.ac.at (A.S.); 2 Institute for Synthetic Bioarchitectures, Department of Nanobiotechnology, University of Natural Resources and Life Sciences, Muthgasse 11, 1190 Vienna, Austria; christoph.zaba@fmc-ag.com

**Keywords:** superparamagnetic iron oxide nanoparticle, cationic magnetopolymersomes, cationic lipopolymersomes, confocal microscopy, transmission electron microscopy, cell-uptake, cytotoxicity

## Abstract

We present a potential theranostic delivery platform based on the amphiphilic diblock copolymer polybutadiene-*block*-poly (ethylene oxide) combining covalent fluorescent labeling and membrane incorporation of superparamagnetic iron oxide nanoparticles for multimodal imaging. A simple self-assembly and labeling approach to create the fluorescent and magnetic vesicles is described. Cell uptake of the densely PEGylated polymer vesicles could be altered by surface modifications that vary surface charge and accessibility of the membrane active species. Cell uptake and cytotoxicity were evaluated by confocal microscopy, transmission electron microscopy, iron content and metabolic assays, utilizing multimodal tracking of membrane fluorophores and nanoparticles. Cationic functionalization of vesicles promoted endocytotic uptake. In particular, incorporation of cationic lipids in the polymersome membrane yielded tremendously increased uptake of polymersomes and magnetopolymersomes without increase in cytotoxicity. Ultrastructure investigations showed that cationic magnetopolymersomes disintegrated upon hydrolysis, including the dissolution of incorporated iron oxide nanoparticles. The presented platform could find future use in theranostic multimodal imaging *in vivo* and magnetically triggered delivery by incorporation of thermorepsonsive amphiphiles that can break the membrane integrity upon magnetic heating via the embedded superparamagnetic nanoparticles.

## 1. Introduction

Polymersomes are biologically inspired nanocontainers formed by self-assembly of amphiphilic block-copolymers in an aqueous environment [1,2]. Such polymersomes are highly appreciated for delivery applications, because their vesicular nature offers convenient transport of hydrophilic, amphiphilic and hydrophobic cargo with improved mechanical and *in vivo* stability compared to existing lipid-based systems [1,3]. Tailoring of the constituent block weights offers tuning of the loading capacity for therapeutic agents and adjustment of permeability characteristics; this makes them particularly attractive for biomedical applications [1,3].

First-generation polymersomes were primarily derived from poly(ethylene glycol) (PEG)-based copolymers because of the excellent water solubility and biocompatibility of PEG [1,4]. Moreover, dense interfacial PEGylation serves to provide self-assembled superstructures with considerable antifouling and stealth properties [4]. The ability to withstand protein adsorption demonstrated for polymer brushes on planar surfaces originates from steric effects that prevent opsonization while stealth properties result from PEG’s non-antigenic character [4]. The resistance to protein adsorption and non-immunicity of PEGylated nanoparticles and vesicles has recently been disputed and its role potentially revised to that of controlling the protein corona rather than preventing protein adsorption [5]. Regardless of mechanism, PEG-based copolymer vesicles confer the well-documented advantages of PEGylated liposome delivery systems to a more stable and versatile system [1]. To these belong low cell-surface recognition due to reduced interactions that trigger internalization by receptor-mediated endocytosis, which in turn yields slow cellular uptake kinetics [6]. Even PEGylated liposomes are often observed to spontaneously fuse to cell membranes. However, cell penetration by fusion via spontaneous mixing of bilayer forming polymers with the lipid cell membrane is unlikely because of the intrinsic high aggregate stability and energetically unfavorable contributions from interfacial line tension that lead to phase separation upon mixing due to the unequal dimensions of block copolymer and lipid [1,3].

Yet, since many anticancer drugs and especially large hydrophilic biopharmaceuticals like enzymes and nucleic acids require intracellular delivery, it is highly beneficial to establish vectors that ensure efficient cellular uptake as well as stable transport in the blood. Endocytotic uptake should be followed by endosomal escape without compromising cell-viability. Commercially employed non-viral vectors mainly rely on cationic modification to increase transfection efficiency [7,8,9] or on polymers that exhibit enhanced affinity with the cellular membrane [6].

Block copolymer architectures offer plenty of opportunities for novel surface functionalization approaches such as functionalization with polymers disruptive to cell-membranes by physisorption or covalent modification, as well as blending with cationic lipids [8,10,11]. Each approach offers its benefits and challenges, which seldom are evaluated in molecular detail. Most frequently, *in vitro* delivery applications are monitored by fluorescence imaging which lacks nanoscopic resolution. Multimodal imaging that employs functional inorganic nanomaterials is an appealing advancement that combines traditional imaging techniques with nanoparticle markers [12]. One such marker, superparamagnetic iron oxide nanoparticles (SPION), is highly compatible with *in vivo* applications, since SPIONs are readily degraded by hydrolysis into their constituent ions and taken up into the body’s iron storage [13,14]. They are employed as negative contrast agents for MRI imaging by their large magnetic moment that reduces relaxation times of protons in the vicinity of the particle [15], but inorganic nanoparticles can also serve as colloidal markers for high-resolution electron microscopy studies. Magnetic probing combined with SPIONs as TEM markers has also been used to investigate nanoparticle organization after cell uptake *in vitro* and *in vivo* [16]. *In vitro* cellular uptake of SPION can be quantified by measuring the iron content of cells [17]. Thus, embedding SPIONs into delivery vehicles such as polymersomes provides the possibility to use multimodal tracking and analysis to investigate the intracellular fate of delivery vehicles across all length scales, from tissue and cell level (MRI, fluorescence microscopy and chemical compositional analysis) to organelle and nanoscale (confocal/super-resolution fluorescence microscopy and transmission electron microscopy) [12]. In addition, the structural incorporation of magnetic nanomaterials into the vesicle membrane allows for switching membrane permeability via magneto-thermal actuation of thermoresponsive membrane components, such as phase separation of lipids or thermoresponsive polymers [18,19,20,21,22,23]. Labeled block copolymer liposomes with embedded SPION are another step towards a theranostic drug delivery system in which both polymer and nanoparticles in addition to multiscale imaging can be tailored to control uptake and trigger release by external or environmental means [12,18,20,24].

We introduce self-assembly of fluorescent polymersomes with membrane-embedded SPIONs that are capable of serving as multimodal and multiscale markers to monitor the intracellular location and degradation of endocytosed delivery vesicles. Various polymersome surface modifications were tested for their potential to promote uptake kinetics and differences in cytotoxicity and endosomal escape behavior.

## 2. Results and Discussion

### 2.1. Preparation of Fluorescent Polymersomes

**Figure 1:**
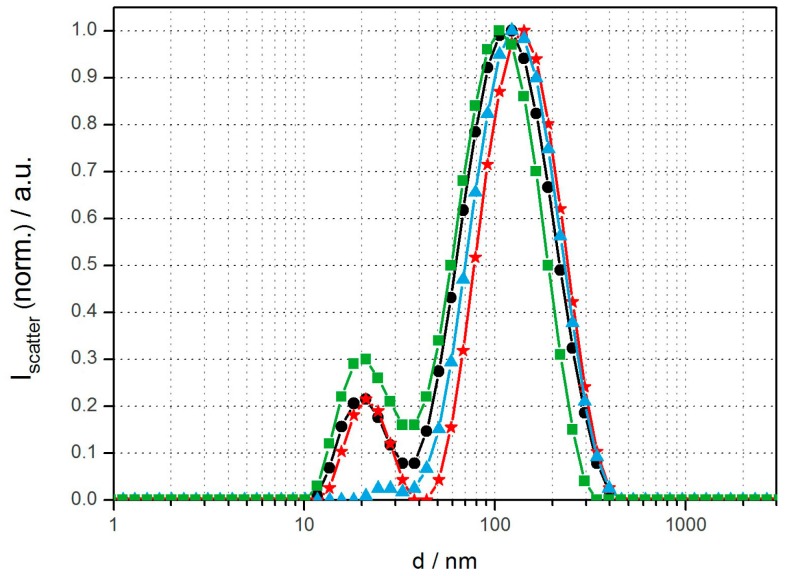
DLS size distributions of various magnetopolymersomes (10% *w/w* SPION) prepared via solvent inversion at 2 mg/mL plus homogenization via extrusion through 100-nm polycarbonate membranes (Avestin): PBD(1200)-*b*-PEO(600)-OH (black), PBD(1200)-*b*-PEO(600)-COOH/*b*-PEI(800) (100% *n/n*) (red), PBD(1200)-*b*-PEO(600)-DEDETA (50% *n/n*) (blue) and PBD(1200)-*b*-PEO(600)-OH/DOEPC (30% *n/n*) (green).

Fluorescent polymer vesicles were prepared by two different methods from the amphiphilic diblock copolymer polybutadiene-*block*-poly (ethylene oxide) (PBD(1200)-*b*-PEO(600), Ð = 1.17). This allowed us to compare the influence of size distribution and particle loading content on cell viability and cell uptake. Rehydration plus extrusion through 100-nm polycarbonate membranes was chosen as an established technique to prepare well-defined unilamellar vesicles with low SPION content. Solvent inversion (THF into water or buffer; 1:10) was used to form vesicles of non-tunable but well-defined size and with high nanoparticle content [25,26]. The obtained size distribution and overall lamellarity of such vesicles varied with aqueous phase composition and polymer concentration, as we have previously described [22,25]. At low amphiphile concentrations (<1 mg/mL) the polymersomes were predominately unilamellar. Concentrations >1 mg/mL resulted in a considerable fraction of multilamellar vesicles, which was determined by TEM and extinction spectroscopy and also previously described in detail [22]. DLS size distributions with intensity size maxima around 20 nm and 100–150 nm in average diameter were obtained for solvent inversion and extrusion with the dominant peak corresponding to vesicles of 150 nm in diameter (Figure 1). This bimodal distribution is attributed to formation of an additional micellar or small unilamellar vesicle (SUV) population coexisting with large unilamellar vesicles (LUV). PBD-*b*-PEO self-assembly is well investigated as one of the original systems used for the spontaneous assembly of block copolymersomes. In the selected size range PBD-*b*-PEO has a very low critical aggregation concentration (CAC) and the assemblies are known to be highly long-term stable, e.g., upon dialysis, with a minute monomer concentration in solution [1,27]. The volume fraction of the hydrophilic PEO and the hydrophobic PBD blocks favors formation of a bilayer membrane in analogy to the theory for amphiphile packing parameters to predict assembled structures [1,21]. However, the polydispersity of block copolymers is large compared to e.g., lipids used to form liposomes; this could explain why a fraction of smaller, possibly micellar, assemblies are often observed in addition to vesicles in in the 100-nm size range. When solvent injection is used to form vesicles, as in one of our approaches, the formation of a less stable fraction of micelles could additionally be aided by the possibility to form micelles or small vesicles at the interface of dissolving small droplets of THF. These smaller assemblies are expected to be in a higher energy state than larger vesicles with less curved membranes; thereby they are out of equilibrium, but due to the mentioned low CAC of PBD-*b*-PEO these structures can be stable over long experimental time scales once they have formed. As delivery systems, micelles and SUVs will have only a fraction of the volume for encapsulating drugs. Therefore, their influence on the sample when investigating drug delivery applications will be negligible.

The obtained vesicle size distributions fall within the limit of clathrin-dependent endocytotic entry of ligand-devoid particles [28]. Without a membrane with stealth properties such particles are expected to be endocytosed. Vesicles were generally formed at 2 mg/mL for cell uptake studies to grant a sufficiently high concentration of vesicles and SPION for reliable *in vitro* experiments.

Modification of PBD-*b*-PEO polymersomes with the fluorophore (7-diethylamino coumarin)-3-carboxyic acid (DEAC-CA) was achieved by Steglich esterification for 3 days in the dark giving 10% dye content. A dye content below 10% is desired to avoid self-quenching [29]. The hydrophobic DEAC acts as fluorophore in membranes of PBD-*b*-PEO without perturbing the block-copolymer physicochemical properties [30]. Its small size minimizes morphological changes of the assemblies caused by the addition of a bulky fluorescent group [31]. DEAC conjugates with PEG are known to be only mildly cytotoxic and possess high quantum yields for the fluorescence emission [32].

Fluorescence spectroscopy on the dye-labeled polymersomes in water exhibited dye emission profiles that are indicative of a low polarity microenvironment (cf. Figure S1). That is, the Stokes shift and quantum yield of the assembled DEAC-copolymer conjugates in water resemble those of the free dye dissolved in a low dielectric solvent (THF) rather than in an aqueous phase (Milli-Q water or buffer). From this we conclude that most of the conjugated dye molecules have self-assembled into the membrane, and that they are located in the hydrophobic membrane interior rather than at the vesicle interface. A loss in overall quantum yield however suggests an equilibrium fraction in contact with water since excited (dialkylamino) coumarins are efficiently quenched in protic, high dielectric solvents by population of twisted intramolecular charge transfer states [33]. No significant change in Zeta potential was detected upon conjugation of DEAC-CA to the PEO-headgroup of the diblock copolymer (see Figure S4). This is expected for a few percent of conjugated entities linked via neutral ester bonds and predominantly located in the membrane interior.

An investigation of vesicle integrity by DLS and fluorescence spectroscopy in environments mimicking relevant cell organelles for degradation (0.5 M PBS and pH = 4.5 for lysosomes, and 1× PBS, pH = 7 and 0.1 µM H_2_O_2_ for peroxisomes) [34,35] showed that PBD-*b*-PEO polymersomes are generally intact and stable with respect to hydrolysis and oxidation after 24 h incubation under those conditions. No changes in the emission profiles or size distributions could be observed during the testing period.

### 2.2. Surface Modification of Polymeric Vesicles

**Scheme 1:**
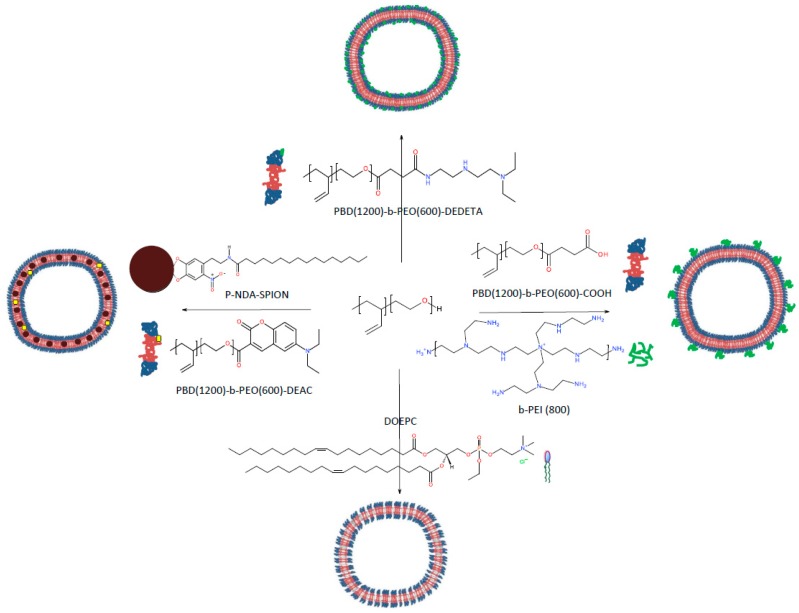
Polymersome surface modifications to promote cell uptake; each can be combined with integration of SPION in the membrane interior.

Various surface modifications of the polymersomes (Scheme 1) were tested for their potential to enhance cell penetration of stealth polymersomes. The tested modifications were surface adsorption of membrane-disruptive low-Mw polymer, covalent modification of the PBD-*b*-PEO with a short oligoamine sequence and a blend of PBD-*b*-PEO and cationic lipid as enhancers to promote cell uptake. Cell uptake was investigated using human cervical adenocarcinoma cells (HeLa) that constitute a robust and often used model system to test promoted uptake, e.g., of transfection vehicles.

**Figure 2:**
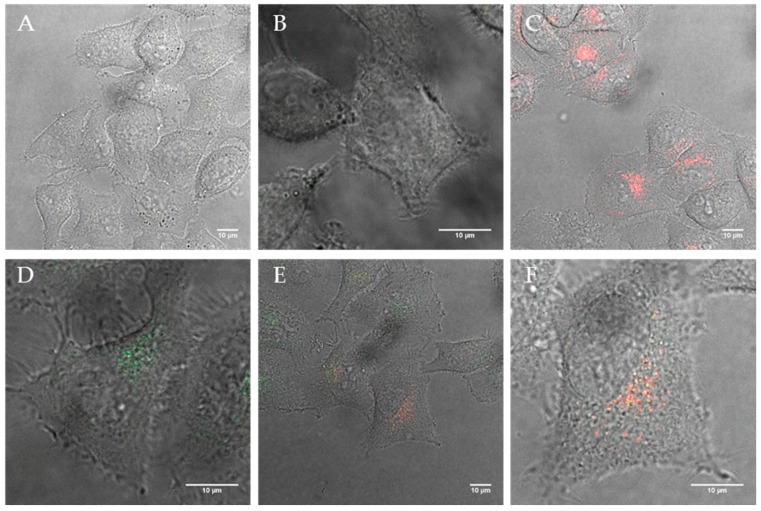
Confocal images of (**A**) HeLa cells (negative control), (**B**) HeLa cells after 12 h incubation with PBD(1200)-*b*-PEO(600) polymersomes (1% DEAC labeled), (**C**) HeLa cells after 12 h incubation with cell light stain expression a red fluorescent protein (RFP) in lysosomes, (**D**) HeLa cells after 12 h incubation with neutrally charged but polycation coated PBD(1200)-*b*-PEO(600)-COOH/*b*-PEI(800) polymersomes (positive control), (**E** + **F**) co-localization of the fluorescently labeled cationically coated polymersomes (green) within lysosomes (red). Unmodified polymersomes exhibit negligible uptake while those modified with cationic *b*-PEI show increased frequency of internalization and localization within lysosomes.

Overnight incubation of HeLa with differently prepared DEAC-labeled polymersomes gave rise to hardly detectable fluorescence within washed cells (Figure 2B). This indicates that PBD-*b*-PEO vesicles exhibit significant stealth properties and slow cellular uptake even when modified with fluorescent tracers; this finding is in line with earlier reports on uptake of PEGylated polymersomes *in vivo* by eukaryotic cell lines, showing that they are essentially not detected by normal cells [4,6]. To achieve significant cell internalization, the polymersome interface must be modified with markers promoting cell uptake.

Branched poly(ethylene imine) or *b*-PEI is a commonly used non-viral transfection agent comprising a combination of primary, secondary and ternary amines that strongly interact with negatively charged molecules such as DNA or native cell membranes [7,8,9]. Adsorption of these membrane-disruptive agents to polymeric delivery vesicles was recently exploited for transfection because the mechanical robustness of the vesicles allows for direct coating without breaking the membrane integrity [10]; this is a distinct advantage over comparable liposome delivery systems. Moreover, *b*-PEI promotes endosomal escape from lytic organelles through an osmotic proton sponge effect, which is essential for active compounds to reach their intracellular target [6,9,10]. Neutral polymersomes require hydrolytic cleavage of the amphiphile forming the bilayer to develop lytic properties through a change of the hydrophilic-hydrophobic balance [36]; that, however, results in slow uptake kinetics when PEG is used as the non-degradable hydrophilic block [36].

We increased the affinity of *b*-PEI to the vesicle surface by carboxylation of the hydrophilic PEO-block with succinic anhydride prior to adsorption of *b*-PEI to the modified vesicles. Near-quantitative end-group modification was verified by ^1^H-NMR and FTIR spectroscopy (see synthesis and characterization section in the supplementary material). The resulting acid-terminated polymer vesicles (−40 mV) exhibited a clear shift in Zeta potential of −36 mV compared to the unmodified hydroxyl-functionalized diblock-copolymer assemblies (−4 mV) in 0.1× PBS (cf. Figure S4). The pH in early endosomes of 5–5.5 is close to the pKa of the carboxylic acid functionalization. Hence, a weakening of the attraction of the polycationic surface coating in the endosome can increase the membrane disruptive and lytic effects of *b*-PEI to facilitate endosome disruption.

Addition of 1 mole-equivalent of *b*-PEI to pre-formed PBD(1200)-*b*-PEO(600)-COOH vesicles only yielded a modest change in Zeta potential independent of adsorption time (1–24 h), while addition of 10× mole-excess drastically increased the surface charge. Subsequent syringe filtration through 0.2-µm PVDF units, however, yielded negative Zeta potentials similar to those obtained for 1 equivalent (eq.) of *b*-PEI (−25 mV). In contrast, purification of the sample via size exclusion chromatography over Sephadex G-75 leads to charge neutralization (−2 mV). This finding implies that the low-Mw *b*-PEI polyelectrolyte is adsorbed in patches to the vesicle surface rather than being quantitatively associated with the entire surface as seen in the case of high-Mw analogues that readily invert surface charge [10].

Zeta potentials of neutral PBD-*b*-PEO polymersomes and those of the PBD-*b*-PEO-COOH/*b*-PEI(800) samples purified by chromatography were almost identical (cf. Figure S4). Despite this, confocal microscopy showed markedly increased cell uptake for the latter after 24 h incubation with HeLa cells (see Figure 2). This is attributed to the direct accessibility of the cationic polymer coating on the vesicle surface, leading to enhanced cell surface recognition.

Sub-cellular co-localization studies were conducted by staining HeLa for specific cell compartments with CellLight^®^ for 20 h. The stain expresses a red-fluorescent protein tag (RFP) fused to a signaling peptide, here lysosomal associated membrane protein 1 (lamp1), which provides specific targeting of cellular lysosomes. 12 h incubation with cationic polymersomes at 300 µg/mL and staining exhibits co-localized fluorescence near the nuclei (red—lysosomes and green—PBD(1200)-*b*-PEO(600)-DEAC in Figure 2). This result implies internalization of polymersomes into lysosomes.

The other alternative we tested for mild surface modification of the vesicles was the covalent attachment of an oligoamine sequence to the hydrophilic COOH-terminated polymer PEO-block. Diethyldiethylenetriamine (DEDETA) is frequently employed as cationic lipid transfection agent. The Mw of the DEDETA fragment is low and thus it is not expected to trigger a significant volume transition that disintegrates the vesicle, but it could provide a membrane-disruptive potential upon charging in the endosome. Based on ^1^H-NMR a total functionalization degree of 70% was obtained. Vesicles of PBD(1200)-*b*-PEO(600)-DEDETA had a Zeta potential of −6 mV compared to −40 mV for the acid-terminated precursor (cf. Figure S4).

**Figure 3:**
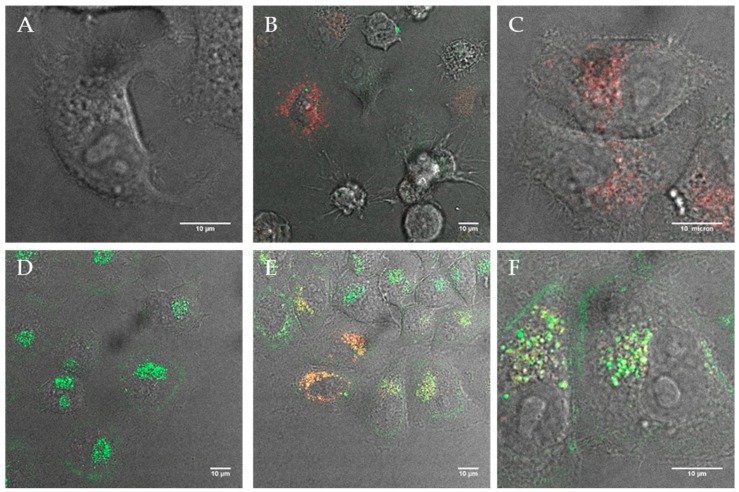
Confocal images of (**A**) HeLa cells after 12 h incubation with polymersomes containing 40% DEDETA (1 % DEAC; green) and (**B** + **C**) the corresponding lysosome (red) co-localization images; Image (**D**) shows HeLa after 12 h uptake with 20% DOEPC-blended lipopolymersomes (green) and (**E** + **F**) show the co-localization within lysosomes (red).

DEDETA-modified vesicles yielded intra-cellular fluorescence comparable to that of unmodified vesicles (Figure 3A–C), which demonstrates ineffective cell uptake compared to *b*-PEI coated vesicles, despite the similar Zeta potential. This suggests that the covalently bonded oligoamine segments are shielded by the hydrophilic corona while *b*-PEI coated on the vesicles is accessible to cellular recognition. Partitioning of a significant fraction of the limited number of cationic end-segments into the PEO shell [37] as well as partial aggregation with remaining unconsumed COO^−^ and OH end segments can be expected. A high Mw polymer like *b*-PEI is likely to remain adsorbed to the surface of the vesicles in patches [37].

A third and elegant approach that avoids surface modification of diblock copolymers and purification is blending the polymeric assembly with cationic lipids [11,38]. This approach is not only thought to largely avoid neutrophil recognition by disguising the lipid antigen underneath a superficial PEG layer but also to promote membrane interactions in a pH-independent way as quaternary ammonium groups are permanently charged. Tuning the cationic lipid fraction allows for tuning presentation of the lipid antigen to the cell surface [11]. Incorporation of a lipid fraction in polymersomes containing magnetic nanoparticles in the hydrophobic membrane core has been reported to yield stable vesicles [22].

We chose 1,2-dioleoyl-*sn*-glycero-3-ethylphosphocholine chloride salt (DOEPC) to achieve a homogeneous lipopolymersome blend. The incorporation of doubly unsaturated lipids was previously shown to give a uniform lipid distribution within PBD-*b*-PEO vesicles of moderate Mw [39]. This homogeneity is supposedly further improved in our case by additional dispersion through charge repulsion among the cationic lipids. The Zeta potential of the blended sample was measured to be overall cationic at +16 mV. The lipid/polymer blend had considerably higher Zeta potential than the other polymer surface modifications, but considerably lower Zeta potential than liposomes prepared from blends of DOEPC and POPC (+50 mV). The incremental change in Zeta potential upon addition of DOEPC to POPC was nearly twice as high as when added to PBD-*b*-PEO. This implies that the lipid headgroups are partially shielded by the hydrated, neutral PEO brush surrounding the polymersome and not directly exposed at the interface as for liposomes [3].

The cationic lipopolymersomes showed the by far highest efficiency for cell uptake (see Figure 3). The subcellular localization of cationic lipopolymersomes paralleled that of *b*-PEI-coated and DEDETA-modified vesicles. A high degree of co-localization with lysosomes was observed, although fluorescence from vesicles in areas without significant fluorescence from the lysosome stain also could be observed (see Figure 3). The much higher net charge is a crucial factor that significantly contributed to the improved uptake of the lipopolymersomes compared to the other cationic vesicles.

### 2.3. Magnetopolymersomes

**Figure 4:**
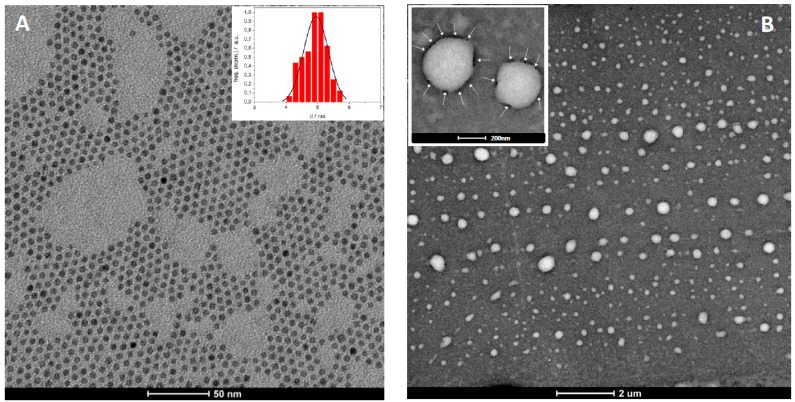
TEM micrographs of (**A**) as-synthesized monodisperse SPION with a size distribution of 5.0 ± 0.4 nm and (**B**) ultra-thin sections of hydrophobic SPION embedded in PBD(1200)-*b*-PEO(600) polymersome membranes prepared by solvent inversion at 0.5 mg/mL amphiphile concentration. The arrows in the inset indicate the locations of SPION in the membrane.

Superparamagnetic iron oxide nanoparticles were prepared through thermal decomposition of Fe(CO)_5_ in the presence of oleic acid to control their size and morphology; the resulting SPION were subjected to a rigorous protocol for ligand exchange to produce hydrophobic SPION irreversibly grafted with a dense shell of palmityl-nitrodopamide (P-NDA), as previously reported [40]. Figure 4A shows TEM micrographs of the synthesized SPION with a narrow size distribution of 5.0 ± 0.4 nm.

**Figure 5:**
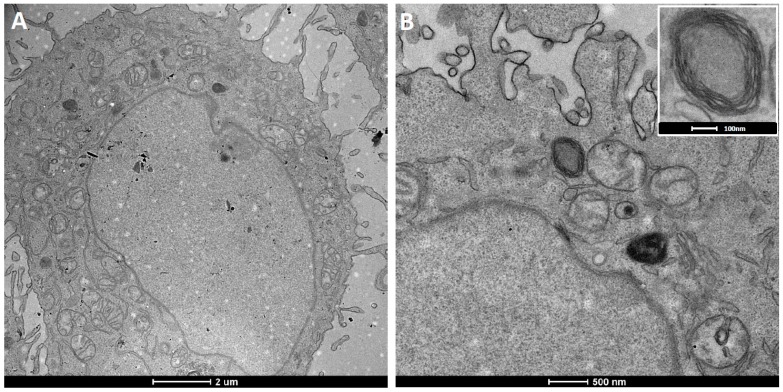
TEM ultrathin sections of HeLa cells after 12 h incubation with fluorescent magnetopolymersomes (PBD(1200)-*b*-PEO(600), 10% SPION, 5% DEAC). (**A**) Overview of a typical preparation showing few polymersomes as dark spherical objects and (**B**) peripheral cell region with a rare observation of an internalized magnetopolymersome. The inset depicts a close-up of a multilamellar magnetopolymersome judged from contrast and membrane thickness.

Successful embedding of hydrophobic SPION at high density in the membrane was achieved using solvent inversion [21,25,41,42]. A high density of SPION per vesicle is crucial to applications as magnetic contrast agents, as well as for triggered drug delivery, for which susceptibility to magnetically triggered release is enhanced in direct proportion to the nanoparticle concentration in the membrane [23,43]. Localization of nanoparticles exclusively in the bilayer region was demonstrated by ultra-thin sectioning of the PBD(1200)-*b*-PEO(600) magnetopolymersomes in Figure 4B. The vesicles are predominately unilamellar at low polymer concentrations (0.5 mg/mL in Figure 4B). Increasingly multilamellar vesicles are observed for samples prepared at higher concentrations (cf. 2 mg/mL in Figure 5) [22]. Polymersomes with SPION in the membrane have previously been suggested and demonstrated as potent magnetic resonance imaging (MRI) contrast agents [12,44]. The high density of SPION we achieve in the membrane can lead to formation of nanoparticle clusters that increase vesicle permeability and magnetic relaxivity [22,45].

Incorporation of SPION into the block copolymer membrane lead to a drastic decrease in DEAC fluorescence intensity by approximately 70%, but the signal is still easily distinguished from the cellular auto-fluorescence background for 10% *w/w* SPION loading (cf. Figure S2). The quenching of DEAC fluorescence is due to non-radiative transfer of the excitation to the nanoparticle core and the nitrocatechol within the Förster radius of the SPION. This was demonstrated by the complete quenching of fluorophores coupled directly to the nanoparticles and therefore residing within a few nm of the SPION core surface. The high SPION density in the membrane ensures similar proximity of the DEAC dye to a SPION acceptor, since DEAC partitions into the membrane interior as shown above.

Cell uptake of polymersomes incorporating SPION by HeLa cells was observed by confocal microscopy analogously to for polymersomes without SPION. Also, a variation of the fraction of SPION incorporated in the membrane was performed in these experiments. This showed that also a high SPION content does not compromise the non-fouling and stealth properties of polymersomes without surface modification in terms of suppressed cell uptake. A negligible interaction with serum proteins was further confirmed by that no associated proteins could be detected by analyzing SPION-loaded polymersomes by electrophoresis after incubation in cell culture media (see Figure S5).

Furthermore, also TEM confirmed low uptake of unmodified magnetopolymersomes with a neutral, non-zwitterionic outermost PEG corona. PBD-*b*-PEO vesicles can be identified in TEM by positive staining of the unsaturated PBD-block with OsO_4_. A rare event, judged from the analysis of a large number of cells, shown in Figure 5 depicts an internalized, multilamellar, SPION-loaded polymersome after 24 h of incubation. Such events were not observed in the negative control, but since it is rare also this observation could be an artifact, e.g., due to staining. The presumed ingested SPION-loaded polymersomes were structurally intact without any signs of decomposition. Cellular ultrastructure was highly conserved (pool of around 100 samples). Importantly, uptake was very low and no indications of excessive apoptosis or necrosis could be detected as consequence of incubation with stealth magnetopolymersomes.

**Figure 6:**
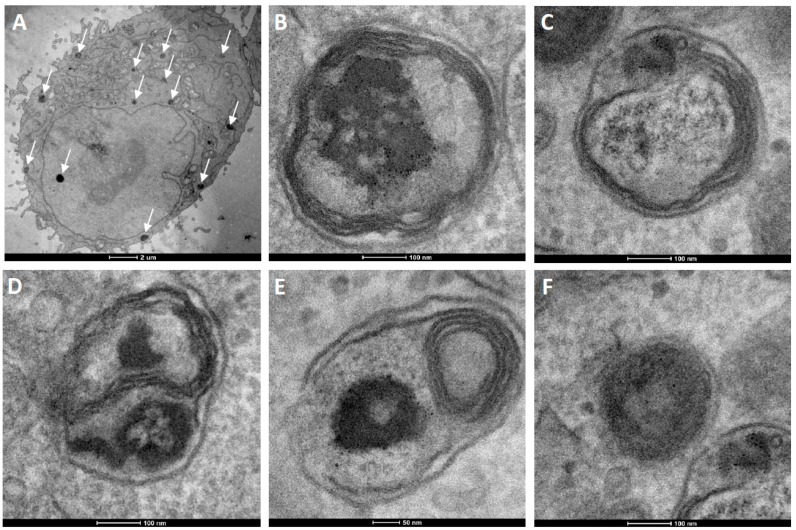
TEM ultrathin sections of HeLa cells after 12 h incubation with cationic fluorescent magnetopolymersomes. Vesicles (50% PBD-*b*-PEO-COOH; 2% DEAC) containing 10% *w/w* SPION were formed at 2 mg/mL via solvent inversion and subsequently coated in 10× *b*-PEI(800) before being purified by gel permeation chromatography. Overview in (**A**) shows elevated levels of cationically modified magnetopolymersomes taken up (black spots indicated by arrows). The sequence (**B**–**F**) shows higher magnification images of the apparent progression of hydrolytic degradation of SPION-loaded polymersomes after internalization.

TEM micrographs of embedded HeLa cells after 24 h of incubation with SPION-loaded PBD(1200)-*b*-PEO(600) vesicles surface-modified with *b*-PEI show enhanced uptake compared to unmodified stealth magnetopolymersomes (see Figure 6). Incipient signs of SPION degradation were also observed. Particle degradation is observed in TEM by the appearance of SPION with increasingly smaller size and broadening size distribution. The advantage of using monodisperse SPION as microcopy markers is that an initially very monomodal distribution centered around 5 nm is observed to become ill-defined with emerging features below 3 nm and homogeneous high contrast areas developed in the vicinity of the polymersome membranes. The latter we tentatively attribute to iron salts associating with the polymer such as iron complexes of PEG. PEG is known for its ability to complex various metals, including ferric ions in a tetrahedral fashion [46]. Complexed metal ions enhance the electron density and therefore TEM contrast, as observed in the micrographs. A strong indication that this indeed was an effect of dissolved and complexed iron ions, is that it was only observed for samples in which SPION were present and dissolved. Therefore, it is not likely to be an artifact from straining, although this cannot be completely ruled out from TEM alone. The localization of cationic vesicles over many hours in late lysosomal compartments observed for these samples thus seemed to cause hydrolysis of the embedded nanoparticles. The lysosome pH of 4.5 has been reported sufficient to decompose bulk magnetite within a similar time [47].

Multilamellar magnetopolymersomes coated with *b*-PEI also showed vesicular disintegration after 24 h. Figure 6B–F depicts a sequence of events recorded at various locations within a cell. It is likely that *b*-PEI enforces a proton gradient that efficiently triggers nanoparticle dissolution and that released decomposition products are responsible for exfoliation of polymer lamellae. A locally high concentration of released iron ions complexing with PEG, as tentatively observed in Figure 6 and reported for dissolved PEGylated SPION [48], could influence polymer conformation and in turn affect the packing parameter of the amphiphile and thereby membrane integrity. The accompanying change in membrane permeability would lead to a further rise in local proton concentration across the membrane and hence autocatalytically accelerate SPION dissolution. Complexation could lead to the apparent separation of polymer layers containing iron ions from the original assembly into segregated compartments when the concentration of iron ions reaches above a threshold value. The segregated compartments seem to excrete from the parent polymersome by budding into individual clusters (see Figure 6). Apparently, making the vesicles cationic promoted not only endosomal uptake, but also both polymersome and SPION degradation in the lysosome. This finding is similar to recent observations of variable degradation rates for iron oxide nanoparticles depending on their surface (polymer) coating in long-term studies *in vitro* and *in vivo* [49,50].

### 2.4. Cytotoxicity and Iron Content

**Figure 7:**
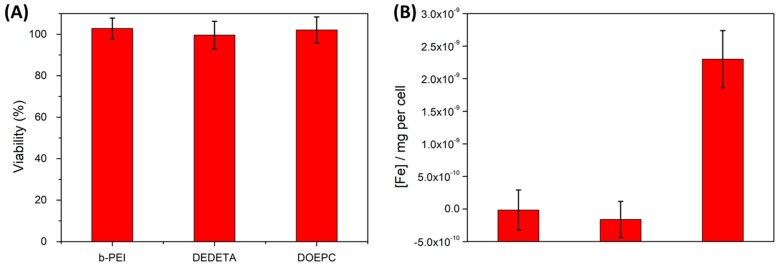
Cytotoxicities (**A**) and iron uptake data (**B**) of HeLa cells after 12 h of incubation with various polymersome preparations comprising the cationic modifications listed in the graphs, at 300 µg/mL amphiphile concentration. Cytotoxicity assays did not show any measurable decrease in cell viability among the different preparations within the accuracy of the method. Iron content data (normalized to the average cellular Fe-background concentration) depicted a significant increase in iron concentration only for lipopolymersomes. PrestoBlue and Ferrozin assays were performed in triplicates.

Cytotoxicities of the different types of magnetopolymersomes were evaluated by a PrestoBlue^®^ assay applied to HeLa cells, which assesses the metabolic activity of the cells [51]. The results are shown in Figure 7A. Additionally, the uptake of magnetopolymersomes with different surface modifications was investigated by determining the iron content of cells exposed to magnetopolymersomes for 24 h using a Ferrozin assay [17].

Previous separate investigations of SPION and oligo(ethylene glycol)-dicoumarin showed very low cytotoxicities for both species [32,52]. SPION toxicity is predominately associated with the catalytic generation of reactive oxygen species (ROS) by Fenton-type reactions upon exposure of the naked nanoparticles to cells. The production of ROS and therefore the cytotoxicity can be reduced by appropriate surface capping strategies [52,53]. However, extremely high intracellular levels of iron can still cause toxicity attributed to free radical generation and high iron levels [52].

Our co-formulation of P-NDA-coated SPION and DEAC-labeled PBD-*b*-PEO copolymer vesicles yielded negligible cytotoxicity at a dose of 300 µg/mL of total polymer mass. An increase in the SPION content to 20% *w/w* showed a minor and statistically insignificant increase in cytotoxicity. This tentative trend was independent of the preparation method (cf. Figure S5). For neutrally charged magnetopolymersomes the absent cytotoxicity can be explained by the negligible uptake of the stealth vesicles demonstrated by fluorescence microscopy, TEM and the Ferrozin quantification of iron uptake (Figure 6B).

Cytotoxicity tests of the different cationic magnetopolymersome preparations similarly exhibited no significant effects on cell viability when HeLa cells were incubated at 300 µg/mL of total polymer mass (Figure 7A). High-Mw cationic polymers were reported to yield full inversion of vesicle surface charge upon adsorption that promotes uptake, but they were also shown to induce significant cytotoxic effects such that their action had to be limited by incubation time [10]. In contrast, the low-Mw *b*-PEI(800) used in this work appears non-cytotoxic even after 24 h incubation, and can be used as non-toxic surface modification of vesicles. Thus, the reduced efficiency of cell uptake is offset by negligible cytotoxicity, i.e., much higher concentrations and longer incubation times can be used.

Covalent surface modification of the magnetopolymersomes with a short cationic oligoamine segment showed no cytotoxicity, but also resulted in no detectable uptake by fluorescence microscopy, TEM or by the quantitative Ferrozin assay.

Magnetolipopolymersomes with membranes composed of cationic lipid DOEPC and PBD-*b*-PEO showed much higher uptake by all methods of investigation, including the Ferrozin test measuring iron uptake. It was the only cationic modification approach to show significantly increased iron levels in the cells by a concentration two times higher than the background control (Figure 7B and Figure S8). These hybrid vesicles combined enhanced uptake with no demonstrable cytotoxicity at 300 µg/mL organic concentration (Figure 7A). We emphasize that using a quantitative assay for iron concentration in the cell that directly corresponds to the uptake per cell of the inorganic nanoparticle marker, we avoid several weaknesses of traditional methods to determine amphiphile vesicle uptake. The vesicle carries the quantitative and non-toxic nanoparticle tag in its interior and we do not require post-labeling of vesicles that have already been taken up by cells. Finding specific labels for vesicle membranes that do not interact with organelles of the cell is very challenging. An interior nanoparticle label can also be applied regardless of membrane functionalization strategy, providing a tool to compare the efficiency of different functionalization methods. Having a label that can only be transported by the vesicular structure into the cell and which is not subject to lipid-lipid or amphiphile-lipid exchange between membranes also ensures certainty that we are quantitatively determining vesicular cell uptake and not just materials transfer. Indeed, this reporter function adds greatly to the multimodal tracking and imaging functions of the fluorescent magnetosomes.

## 3. Materials and Methods

### 3.1. Materials

All chemicals and cell culture media were purchased from Sigma Aldrich (Vienna, Austria). Assay reagents were obtained from Sigma Aldrich and Carl Roth (Vienna, Austria). CellLight^®^ Lysosomes-RFP BacMam 2.0 was supplied by Thermo Fisher Scientific (Vienna, Austria).

The synthesis of monodisperse, N-palmityl-6-nitrodopamide coated superparamagnetic iron oxide nanoparticles (P-NDA-SPION) with a tunable core diameter was described previously [40].

Polybutadiene-*block*-poly (ethylene oxide), PBD(1200)-*b*-PEO(600) (Mw = 1800 g/mol, Ð = 1.17) was obtained from Polymer Source Inc. (Montreal, QC, Canada) and used as received.

1,2-dioleoyl-*sn*-glycero-3-ethylphosphocholine chloride salt (DOEPC) was purchased from Avanti Lipids Inc. (Alabaster, AL, USA) and used without further purification.

### 3.2. Methods

TEM: TEM studies were performed on a FEI Tecnai G2 20 transmission electron microscope (Brno, Czech Republic) operating at 160 kV. Ultrathin sections were prepared using a Leica Ultracut UC-7 (Leica Microsystems Inc., Buffalo Grove, IL, USA) equipped with a Diatome Ultra 45° diamond knife (DiATOME, Hatfield, PA, USA). 70-nm slices of fixed and embedded cells were transferred onto 150 mesh hexagonal copper grids coated with Pioloform. After air-drying, samples were investigated without further staining.

Confocal Microscopy: All images were recorded on a Leica TCS SP8 Laser Scanning Confocal Microscope (Leica Microsystems Inc., Wetzlar, Germany) equipped with LCS software and a HC PL APO 100×/1.40 oil STED white objective. Cells were imaged in transmission mode after seeding 1 mL of cell suspension (~2.5 × 10^5^ cells/mL) in confocal dishes (In-Vitro Scientific, Sunnyvale, CA, USA) for 24 h. The cells were incubated with fluorescent magnetopolymersomes at 300 µg/mL for 12 h. For co-localization studies HeLa cells were further stained with CellLight^®^ lysosome-RFP (Thermo Fisher Scientific, Vienna, Austria) by incubation for 6 h. Samples were excited at 405 nm (DEAC) or 560 nm (RFP) and their emissions were collected at 420–530 nm (DEAC) and 580–715 nm (RFP). All samples were imaged at room temperature. All spectra were background corrected for autofluorescence of the cells.

Dynamic Light Scattering: Hydrodynamic diameters and Zeta potentials were recorded on a Malvern Zetasizer Nano-ZS (Malvern Instruments, Malvern, UK) in PBS (10 mM NaHPO_4_, 2.7 mM KCl, 137 mM NaCl, pH = 7.4) at 25 °C in 173° backscattering mode. Samples were equilibrated for 120 s each and the autocorrelation function was obtained by averaging 3 runs. Samples were diluted 1:10 with Milli-Q for Zeta potential measurements.

### 3.3. Synthesis

All polymer modifications were conducted under inert atmosphere using standard reaction procedures. The crude reaction mixtures were purified by liquid-liquid extraction and flash column chromatography over silica (see synthesis section in the Supplementary Material for details on the preparations and characterization).

### 3.4. Sample Preparation

Detailed descriptions of the sample preparation are found in the Supplementary Material. In brief:

Vesicle suspensions: Magnetopolymersomes were formed by assembling the respective mixtures via solvent inversion (THF into H_2_O; 1:10) at 0.5–2 mg/mL total amphiphile concentration similar to previously published protocols [25]. Samples were sonicated for 30 min at room temperature on a Transsonic T 460 bath before the organic solvent was evaporated for several hours under a gentle stream of nitrogen gas. *b*-PEI coated samples for cell uptake were prepared by forming residue-free magnetopolymersomes as described above, followed by coating with a 10-molar excess of *b*-PEI(800) under magnetic stirring. After an adsorption period of 1 h the samples were purified by gel filtration over Sephadex G-75 (GE Healthcare Life Sciences, Vienna, Austria).

Cell culture: Human cervical adenocarcinoma cells (HeLa; ACC 57) were obtained from DSMZ (Leibniz Institute DSMZ—German Collection of Microorganisms and Cell Cultures GmbH, Braunschweig, Germany) and grown in RPMI-1640 medium supplemented with 10% (*v/v*) heat-inactivated fetal calf serum (Gibco), 2 mM l-glutamine + 1%: HEPES buffer, antibiotics and amino acids in humidified 5% CO_2_ atmosphere at 37 °C.

Ultrathin-sections: Embedding of cells was conducted in Epon according to a modified protocol of Glauert and Lewis [54]. Briefly, after uptake HeLa cells were washed twice in PBS and fixed in a solution of 2.5% glutardialdehyde, 2.5% paraformaldehyde, 2.5 mM CaCl_2_ and 1% tannic acid in 0.1 M sodium cacodylate buffer (pH 7.4) for 4 h. Fixation was repeated with a fixative without tannic acid for 20 h at 4 °C. After washing in sodium cacodylate followed by distilled water, cells were post-fixed with 1% osmium tetroxide and 1.5% potassium hexacyanoferrate(III) in water for 1 h followed by 2% OsO_4_ in water for additional 2 h at room temperature. After brief washing in water, cells were dehydrated using a graded ethanol series in water (70% − 80% − 90% − 2 × 100%) for 10 min each. After 10 min incubation in propylene oxide, cells were infiltrated with 30%, 60% and 100% epon in propylene oxide for 2 h each. Overnight, cells were incubated with fresh 100% epon at room temperature. The resin was removed and epon mixed with 1.5% accelerator (DMP-30) was added. After 2 h incubation, samples were transferred into gelatin capsules size 00 and filled up with epon/DMP-30. Blocks were cured at 60 °C for minimum 30 h and stored at room temperature.

## 4. Conclusions

We introduced a vesicle platform that allows for multimodal tracking and analysis of the intracellular fate of delivery vehicles across several length scales by combining optical imaging with nanoparticles as markers for ultrastructure research. Fluorescent modification of the polymeric vesicles was achieved by covalent coupling of a non-perturbing coumarin surrogate that is located predominantly in the membrane interior affecting neither cell recognition nor cytotoxicity. High-density co-encapsulation of hydrophobic iron oxide nanoparticles into the polymersome bilayer resulted in considerable fluorescence decrease, but importantly it made it possible to use the SPION as nanoscopic markers for electron microscopy to determine cellular fate and degradation. In future theranostic applications it has the further advantage of rendering the vesicles MRI active [12,18,20] and to enable external control over release of encapsulated compounds by magnetic fields [18,19,20,21,22].

PBD-*b*-PEO-based magnetopolymersomes showed near complete stealth properties in cultures of human cervical adenocarcinoma cells due to their dense PEGylation and their mechanical stability that vastly outperforms PEG-liposomes. The negligible cell uptake predictably led to perfect cell viability. Interaction with the eukaryotic cell surface and cell uptake was adjusted by several mild surface modification approaches, adding known cationic transfection agents to the polymersome membrane. For example, adsorption of membrane disruptive low-Mw, branched poly (ethylene imine) to the polymersomes led to apparently charge neutral magnetopolymersomes with visually enhanced cell uptake and no effect on cell viability.

Co-localization studies by fluorescence microscopy and TEM showed that cationically surface-modified vesicles were preferentially located in lysosomes, which is consistent with an endosomal uptake pathway. Magnetopolymersome stability in endosomal compartments, however, seemed influenced by the presence of cationic agents. Multilamellar, *b*-PEI-coated vesicles showed exfoliation and segregation of metal-rich polymer lamellae. Such destabilization of the delivery vehicle and concomitant release of cargo is a requirement for intracellular delivery of therapeutic agents. However, it also led to dissolution of incorporated SPION that was possibly linked to the exfoliation by metal ion complexation.

The most efficient cell uptake was observed for magnetopolymersomes with a co-assembled blend of PBD-*b*-PEO and DOEPC cationic lipid. These lipopolymersomes had low positive zeta potential with the lipid headgroup charge shielded by the PEG shell and combined efficient uptake with perfect cell viability, as evidenced by fluorescence microscopy, TEM and quantification of iron uptake by the cells. With negligible cytotoxicity, these hybrid vesicles containing SPION as electron microscopy and magnetic contrast agents were both the simplest to assemble and the most efficient platform investigated in this work.

In summary, we have investigated the synthesis, assembly and cell interaction of multifunctional polymersomes. The combination in one platform of the ability to tune cell interactions, fluorescence, TEM markers and the implied ability to perform *in vivo* medical imaging and controlled release exploiting the incorporated but shielded magnetic nanoparticles offers a versatile theranostic platform for further development.

## Supplementary Materials

Detailed synthetic protocols, characterization of compounds and data additional to that reported in the manuscript can be found in the Supporting Material. The following are available online at www.mdpi.com/1996-1944/10/11/1303/s1, Figure S1: Normalized absorption and emission curves of DEAC-CA (left) and PBD(1200)-*b*-PEO(600)-DEAC (right) in various solvents, Figure S2: Normalized absorption and emission curves of PBD(1200)-*b*-PEO(600)-DEAC polymersomes with and without 10% *w*/*w* SPION loading, Figure S3: Confocal images of control magnetopolymersomes prepared via rehydration plus extrusion (100 nm) at 2 mg/mL, Figure S4: Zeta potential distributions of various magnetopolymersomes (10% *w*/*w* SPION) prepared at 5 mg/mL in 1× PBS via rehydration and extrusion through 100 nm polycarbonate membranes (Avestin). Samples were diluted 1:10 with Milli-Q water for measurements, Figure S5: Gel electrophoresis characterization of the supernatants before and after incubation with magnetopolymerosmes, Figure S6: Cytotoxicity data of fluorescent magnetopolymersomes (1% DEAC, 10% SPION) prepared via solvent inversion at 2 mg/mL and by rehydration plus extrusion (31×, 100 nm polycarbonate membrane). Samples were incubated with HeLa cell lines for 12 h before evaluated by Resazurin assays. Cell viability is evaluated as emission ratio 590/560, Figure S7: Data chart for iron uptake of cationic magnetopolymerosmes (10% SPION) as evaluated by Ferrozin assays (C-cellular background, PEI–*b*-PEI modified, poly–DEDETA modified, lipid–DOEPC modified PBD(1200)-*b*-PEO(600) polymersomes). Samples were prepared via solvent inversion at 2 mg/mL and homogenized by post-extrusion (10×, 100 nm).

## Abbreviations

*b*-PEI	*branched*-poly(ethylene imine)
DEAC	7-(diethylamino)coumarin
DEDETA	*N*,*N*-diethyldiethylenetriamine
DOEPC	1,2-dioleoyl-*sn*-glycero-3-ethylphosphocholine chloride salt
HeLa	human cervical adenocarcinoma cells
PBD-*b*-PEO	polybutadiene-*block*-poly(ethylene oxide)
SPION	superparamagnetic iron oxide nanoparticle
