# Supplementary material for: Fluorescent Magnetopolymersomes: A Theranostic Platform to Track Intracellular Delivery
Source: Materials (Basel). 2017 Nov 13;10(11):1303. doi: 10.3390/ma10111303 (PMC5706250; doi:10.3390/ma10111303)
Supplement: Supplementary file 1 [file materials-10-01303-s001.pdf]

Supporting Material

Fluorescent magnetopolymersomes: a theranostic platform to track intracellular delivery

Oliver Bixner[†], Noga Gal[†], Christoph Zaba[§], Andrea Scheberl[†] and Erik Reimhult[†,*]

[†] University of Natural Resources and Life Sciences, Department of Nanobiotechnology, Institute for Biologically Inspired Materials, Muthgasse 11, 1190 Vienna, Austria

[§] University of Natural Resources and Life Sciences, Department of Nanobiotechnology, Institute for Synthetic Bioarchitectures, Muthgasse 11, 1190 Vienna, Austria

* corresponding author: erik.reimhult@boku.ac.at

Reagents

2,2-Dimethyl-1,3-dioxane-4,6-dione 98%; 4-(Diethylamino)salicylaldehyde 98%; Piperidine ReagentPlus 99%; Glacial acetic acid ACS reagent >99.7%; 1,4-Diazabicyclo[2.2.2]octane ReagentPlus ≥99%; Succinic anhydride >99% (GC); Dicyclohexylcarbodiimide puriss ≥99% (GC); 4-(Dimethylamino)pyridine ReagentPlus ≥99; *N,N*-Diisopropylethylamine ReagentPlus ≥99%; *N,N*-Diethyldiethylenetriamine 98%; Branched Poly(ethylene imine) ($<M_w>$ ~ 800 g/mol by LS, $<M_n>$ ~ 600 g/mol by GPC); RPMI-1640 Media; Fetal Bovine Serum (heat inactivated); L-Glutamine Reagent Plus >99% (HPLC); 4-(2-Hydroxyethyl)piperazine-1-ethanesulfonic acid BioPerformance Certified ≥99.5% (titration, cell culture tested); Glutaraldehyde Solution Grade I 25% in H2O (for electron microscopy); Paraformaldehyde Reagent Grade (crystalline); Tannic Acid ACS Reagent; Calcium Chloride Dihydrate ACS Reagent >99%; Sodium Cacodylate Trihydrate BioXtra ≥98%; Potassium Hexacyanoferrate(III) Reagent Plus ~99%; Osmium Tetroxide Solution 2% in H2O (for electron microscopy); (±)-Propylene Oxide Reagent Plus ≥99%; Epoxy Embedding Medium Kit; 2,4,6-Tris(dimethylaminomethyl)phenol >95% (NT) Epoxy Embedding Medium Accelerator; Phosphate buffered saline tablets (0.01 M phosphate buffer, 0.0027 M potassium chloride and 0.137 M sodium chloride, pH 7.4), Milli-Q water (Millipore USA; R=18MΩcm); Ethanol anhydrous ≥99.8%; Acetone Chromasolv Plus for HPLC ≥99.9%; Dichloromethane anhydrous ≥99.8% (contains 40-150ppm amylene as stabilizer); Tetrahydrofuran Chromasolv Plus for HPLC ≥99.9% (inhibitor-free);

Reagents for fixation and dehydration were purchased from Sigma Aldrich. For infiltration and embedding, the Epoxy-embedding kit from Fluka was used. Blocks were formed using gelatin capsules size 00 (Agar Scientific).

Methods

^{1}H- NMR measurements: ^{1}H -solution spectra were collected on a Bruker DPX spectrometer operating at 300MHz. Chemical shifts were recorded in ppm and referenced to residual protonated solvent ($CDCl_3$: 7.26 ppm (^{1}H).

ESI-MS measurements: Mass spectra were collected using a Q-Tof Ultima ESI (Waters, USA) mass spectrometer in positive ion mode (range 100-1500 Da). Samples were dissolved in MeOH and diluted to 100µg/ml.

ATR-FTIR measurements: Mid-IR powder spectra of the lyophilized samples were collected using a Bruker Tensor 37 FTIR spectrometer with a Bruker Platinum Diamond single reflection ATR equipment at a resolution of 4cm^{-1} by averaging 32 scans.

UV-Vis measurements: UV-Vis absorption spectra were collected at a scan speed of 400nm/min on a Hitachi UV-2900 spectrophotometer.

Fluorescence measurements: Fluorescence spectra were collected with a PerkinElmer LS 55 luminescence spectrometer with a scan speed of 400nm/min and a slit width of 2.5 nm.

TGA/DSC measurements: Thermograms were recorded on a Mettler-Toledo TGA/DSC 1 STAR System in the temperature range 25-650°C with a ramp of 10K/min under 80mL/min synthetic air gas flow. The mass loss was evaluated by horizontal step setting.

Synthesis & Characterization

N-palmityl-6-nitrodopamide capped superparamagnetic iron oxide nanoparticles (P-NDA SPIONs)

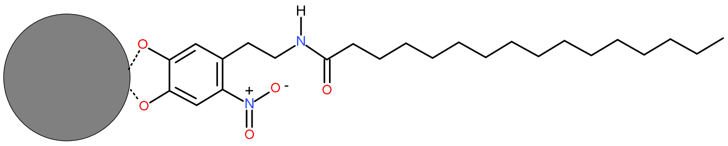

Oleic acid coated magnetite nanoparticles were prepared according to Hyeon et al via thermal decomposition of iron pentacarbonyl in hot surfactant solution.[1] The as-synthesized particles were purified by repeated precipitation from minimal toluene into excess ethanol.

N-palmityl-6-nitrodopamide was synthesized via COMU mediated peptide coupling according to Bixner et al.[2]

Ligand exchange and purification of the particles was conducted as in Bixner et al.[2] The coating exchange was performed in CHCl3:DMF:MeOH=6/3/1 by sonicating equal weight amounts of ligand and as-synthesized SPION under inert atmosphere for 3h. The crude mixture was evaporated to the DMF fraction and precipitated in MeOH. The sample was washed thrice with hot MeOH and collected via magnetic precipitation. Post-coating was conducted in minimal 2,6-lutidine in an excess of ligand at 50°C under nitrogen gas for 48h. P-NDA coated SPIONs were purified by repeated precipitation from hot MeOH.

ATR-FTIR (cm^{-1}): 3600-3000 (b; CONH, OH), 2955 (CH_3), 2921 (CH_2), 2851 (CH_2), 1632 (CONH), 1546 (CONH), 1492 (C=C, NO_2), 1468 (CH_2), 1437 (C=C), 1374 (CH_2), 1320 (NO_2), 1276 (C=C, CO), 1226, 1186, 1117, 1098, 1048 (CO), 880 (PhH), 814 (PhH), 571 (Fe_3O_4), 385 (Fe_3O_4)

TGA (O_2, %w/w): -32; ρ^{graft}= 2.8/nm²

7-(diethylamino)-coumarin-3-carboxylic acid (DEAC-CA)

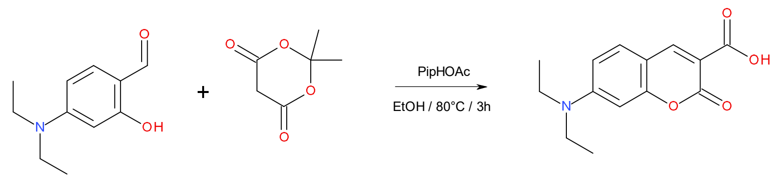

DEAC-CA was prepared by Knoevenagel condensation of *para*-substituted *ortho*-hydroxybenzaldehyde with *alpha*-C-H acidic Meldrum's acid according to Song et al. [3]
In brief, a mixture of 4-(diethylamino)salicylaldehyde (20 mmol), Meldrum's acid (2,2-dimethyl-1,3-dioxane-4,6-dione; 2.89 g, 20 mmol), piperidinium acetate (58 mg, 0.4 mmol) and ethanol (10 mL) was stirred at room temperature for 30 min and refluxed for 3h. The reaction mixture was allowed to cool down to room temperature, followed by chilling in an ice bath for 1h. The product was filtered, washed three times with and recrystallized from EtOH. DEAC-CA was obtained as bright orange crystals in ~ *85%* yield.

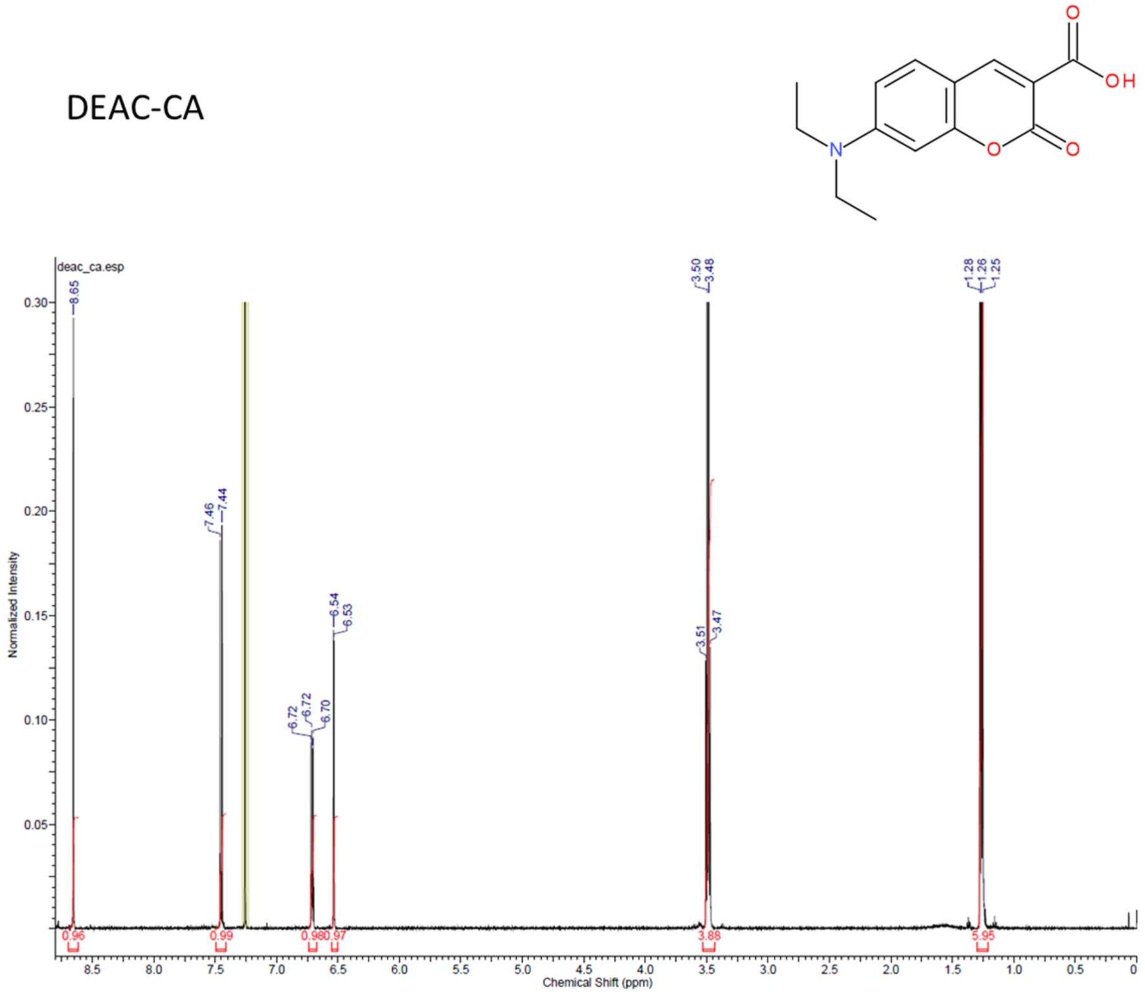

^{1}H-NMR ($CDCl_3$, 300 MHz, ppm): 8.65 (s, 1H, Ph-CH=C), 7.46 (d, 1H, Ph), 6.72 (dd, 1H, Ph), 6.54 (d, 1H, Ph), 3.50 (q, 4H, CH_2), 1.26 (t, 6H, CH_3)

DEAC-CA

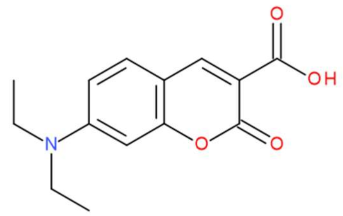

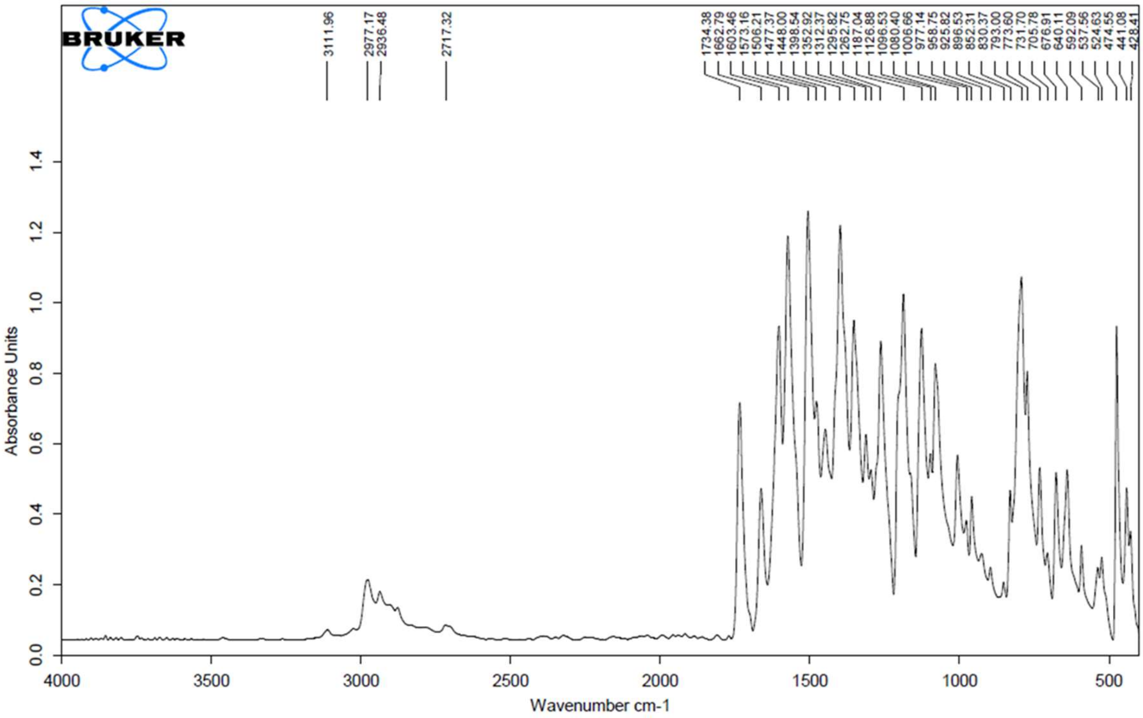

ESI-MS (MeOH, m/z): [M]H$^+$ = 262.11, calc. 262.10; [M]Na$^+$ = 284.10, calc. 284.08

UV/VIS (MeOH, nm): 217, 259 (sh), 423

Emission (MeOH, nm): 482 (λ_{exc} = 420)

Poly(butadiene(1200)-block-ethyleneoxide(600))-O-(7-(diethylamino)-coumarin-3-carboxylic ester) (PBD-b-PEO-DEAC)

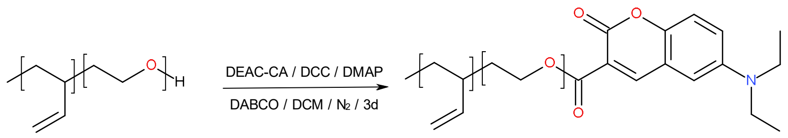

100mg PBD-b-PEO were dissolved in 10ml N_2-saturated, anhydrous CH_2Cl_2 (DCM) under sonication. and subsequently activated for 15 min with 1eq. of 1,4-Diazabicyclo[2.2.2]octane (DABCO). Next, 1.5eq. of 7-(Diethylamino)-coumarin-3-carboxylic acid (DEAC-CA) and 0.2eq. 4-Dimethylaminopyridine (DMAP) were added and the 10% polymer solution was purged with N_2 gas for 15min before cooling to 0°C in an ice-bath. N,N-Dicylcohexylcarbodiimide (DCC, 1.7eq) in 5ml DCM was dropwise added to the magnetically stirred polymer solution at 0°C. The reaction mixture was allowed to slowly warm to room-temperature and reacted in the dark for 3 days under inert atmosphere.
The crude reaction mixture was reduced in volume to approx. 5ml, cooled to -20°C and precipitated DCU was filtered off. The filtrate was diluted with DCM, extracted thrice with 1M HCl, 5% $NaHCO_3$ and washed with Milli-Q water and brine. The combined organic phases were dried over Na_2SO_4, and the cooling-filtration procedure was repeated from minimal acetonitrile (MeCN). The organic phase was loaded onto a SiO_2-column (Silica 60) and washed with several volumes of MeCN to remove excess dye and by-products. The fluorescently labeled target compound was finally eluted in THF:MeOH=10:1. Lyophilization from THF:Milli-Q (1:10) yielded PBD-b-PEO-DEAC as a yellow viscous residue (functionalization approx. 10%).

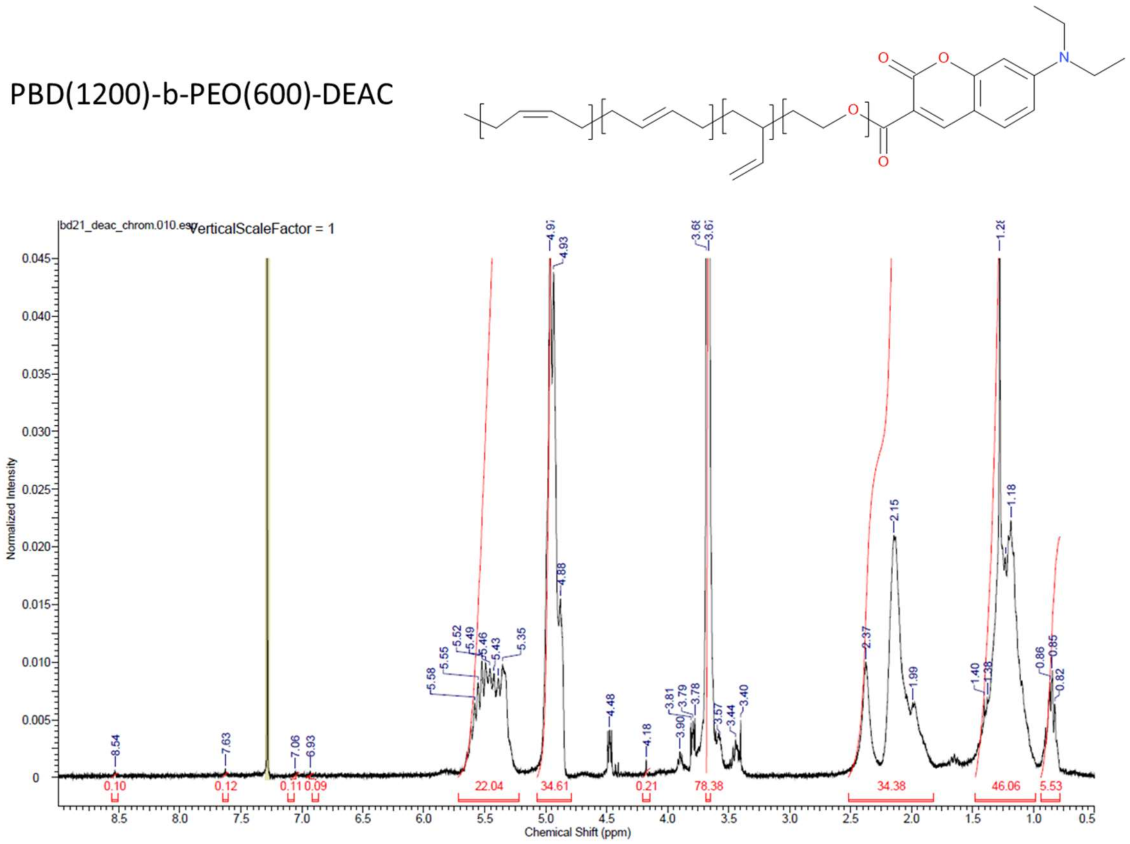

^{1}H-NMR ($CDCl_3$, 300 MHz, ppm): 8.54 (s, 0.1H, Ph-CH=C DEAC), 7.63 (d, 0.1H, PhH DEAC), 7.06 (dd, 0.1H, PhH DEAC), 6.93 (d, 0.1H, PhH DEAC), 5.45 (m, 24H, -C=CH- 1,2-BD, -HC=CH- c,t-1,4-BD), 4.94 (t, 40H, H_2C=C- 1,2-BD), 4.19 (t, 0.2H, -CH_2COO-), 3.66 (m, 60H, -CH_2OCH_2- EG), 2.25-1.8 (m, 28H, -CH_2CH- 1,2-BD, -H_2C-C=C c,t-1,4-BD), 1.5-1.0 (m, 40H, -CH_2CH- 1,2-BD & - alkyl-CH_2 & DEAC-Me_2), 0.85 (t, 3H, alkyl-Me)

PBD(1200)-b-PEO(600)-DEAC

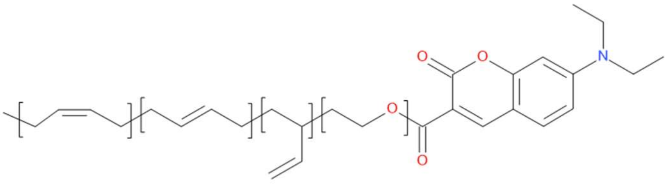

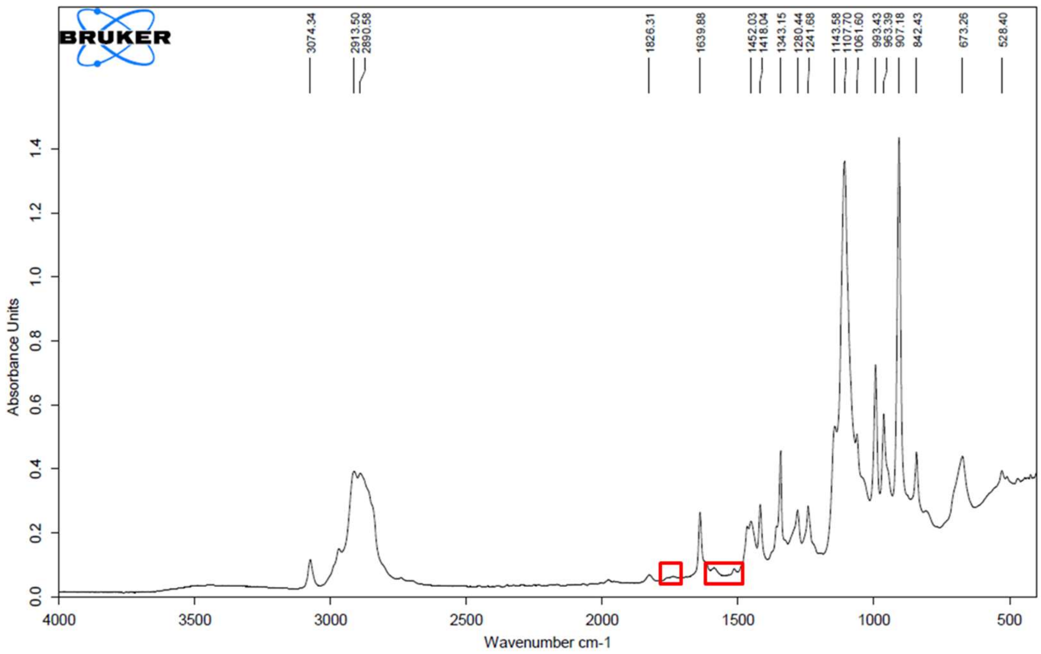

ATR-FTIR (powder, cm^{-1}): 3074 (=CH BD), 2972 (CH_2 BD), 2913 (CH_2 BD), 2890 (CH_2 EG), 1826 (comb.), 1735 (COOR), 1640 (C=C BD), 1622 (C=C aromatic), 1589 (C=C aromatic), 1514 (C=C aromatic), 1452 (CH_2 BD & EG), 1418 (CH_2 BD), 1343 (CH_2 EG), 1280 (CH_2 BD), 1241 (CH_2 BD), 1143 (C-C & C-O EG), 1107 (C-C & C-O EG), 1061 (C-C & C-O EG), 993 (=CH BD), 963 (CH_2 EG), 907 (=CH BD), 842 (CH_2 EG), 673, 528

UV/VIS (MeOH, nm): 223, 259 (sh), 420

Emission (MeOH, nm): 474 (λ_{exc} = 420)

Polybutadiene(1200)-block-polyethyleneoxide(600)-carboxylic acid (PBD-b-PEO-COOH)

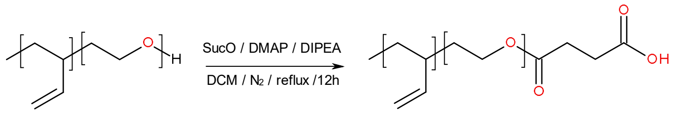

100mg PBD-b-PEO were dissolved in 10ml CH_2Cl_2 (DCM) under sonication and activated with 1eq. of 1,4-Diazabicyclo[2.2.2]octane (DABCO) for 15 min. 0.2eq 4-Dimethylaminopyridine (DMAP) and 3eq succinic anhydride (SucO) in 2ml DCM were dropwise added to the above solution and purged with N_2 for 10min. The reaction mixture was refluxed overnight under inert atmosphere.

The crude product was diluted with DCM, extracted thrice with 1M HCl, 5% $NaHCO_3$, washed with Milli-Q and brine. The organic phases were dried over Na_2SO_4, evaporated and dried in high vacuum overnight to yield ~ 95 % of a transparent viscous residue (functionalization approx. 75%).

PBD(1200)-b-PEO(600)-COOH

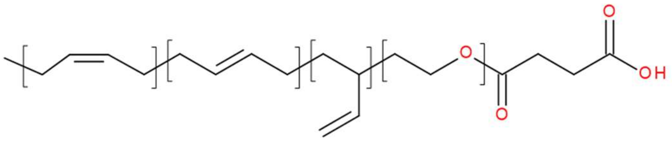

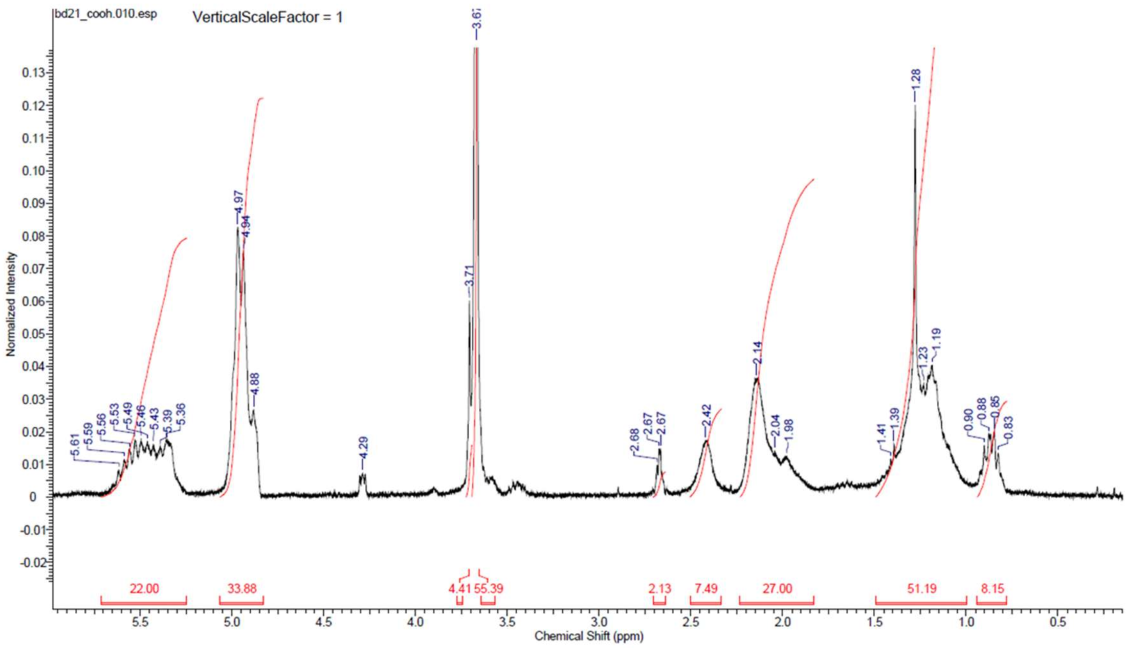

^{1}H-NMR ($CDCl_3$, 300 MHz, ppm): 5.45 (m, 24H, -C=CH- 1,2-BD, -HC=CH- c,t-1,4-BD), 4.94 (t, 40H, H_2C=C- 1,2-BD), 4.29 (t, 1.55H, -CH_2COO-), 3.66 (m, 60H, -CH_2OCH_2- EG), 2.67 (t, 2.95H, -OOCCH_2CH_2COOH), 2.5-1.8 (m, 28H, -CH_2CH- 1,2-BD, -H_2C-C=C c,t-1,4-BD), 1.5-1.0 (m, 40H, -CH_2CH- 1,2-BD & alkyl-CH_2), 0.85 (t, 3H, alkyl-Me)

PBD(1200)-b-PEO(600)-COOH

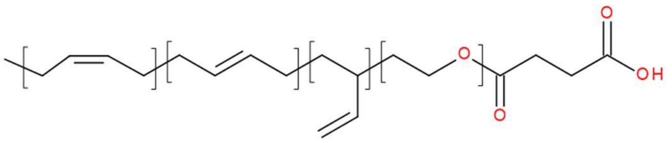

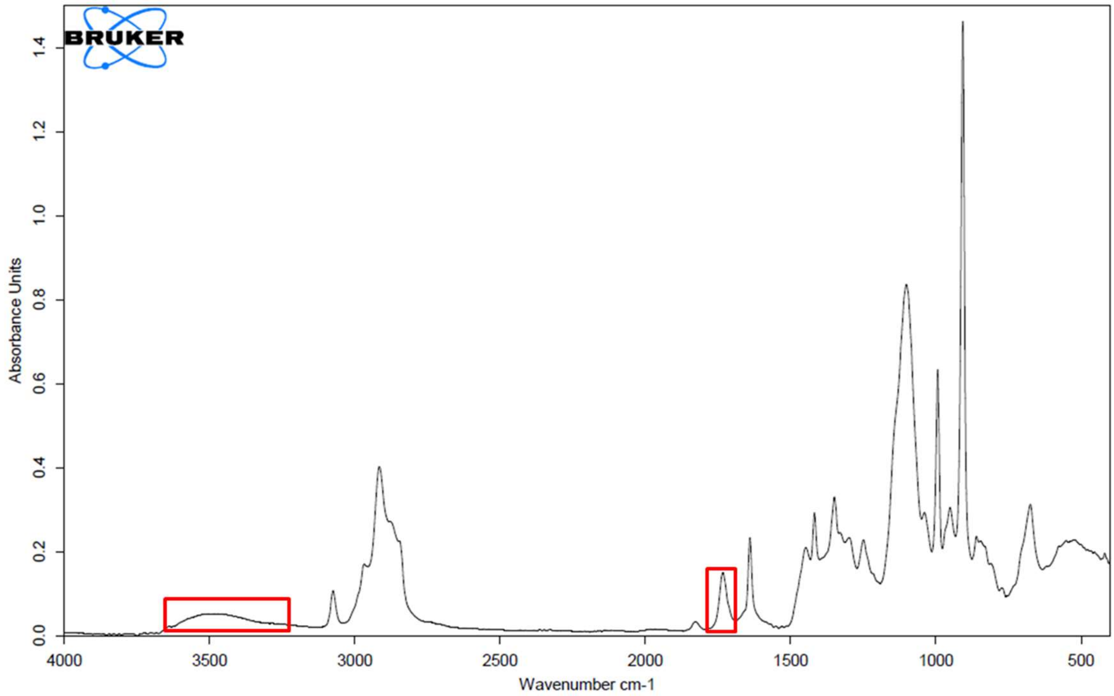

ATR-FTIR (powder, cm^{-1}): 3680-3300 (b, COOH), 2972 (CH_2 BD), 2913 (CH_2 BD), 2890 (CH_2 EG), 1826 (comb.), 1735 (COOR), 1640 (C=C BD), 1447 (CH_2 BD & EG), 1418 (CH_2 BD), 1349 (CH_2 EG), 1330 (COOR), 1300 (COOR), 1249 (CH_2 BD), 1101 (C-C & C-O EG), 1039 (C-C & C-O EG), 993 (=CH BD), 951 (CH_2 EG), 907 (=CH BD), 860 (CH_2 EG), 774, 675, 522

Polybutadiene(1200)-block-polyethyleneoxide(600)-N-{2-[[2-(diethylamino)ethyl]amino]ethaneamide} (PBD-b-PEO-DEDETA)

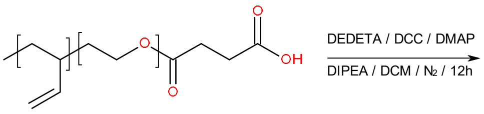

100mg of the above reacted PBD-b-PEO-COOH were dissolved in 10ml CH_2Cl_2 (DCM) under sonication at room temperature before 1eq. 4-Dimethylaminopyridine (DMAP) and 1.5eq of N,N-Diethyldiethylenetriamine (DEDETA) in 1ml CH_2Cl_2 were added. The stirred solution was cooled to 0°C in an ice-bath before 1.2eq of Dicyclohexylcarbodiimide (DCC) in 2ml CH_2Cl_2 were added dropwise. The reaction mixture was slowly allowed to warm to room temperature and reacted overnight under inert atmosphere.

The crude product was reduced to approx. 5ml, cooled to -20°C and filtered to remove precipitated DCU. The filtrate was diluted with DCM, extracted thrice with 1M HCl, 5% $NaHCO_3$ and washed with Milli-Q and brine. The organic phases were pre-dried over Na_2SO_4, evaporated and filtered from minimal acetonitrile (MeCN). The resulting product was purified by flash chromatography over SiO_2 in MeCN and eluted in THF:MeOH=10:1. After drying in high vacuum overnight PBD-b-PEO-DEDETA was obtained in ~ 90% yield as a transparent to off-white viscous residue (conversion approx. 95%; functionalization approx. 70%).

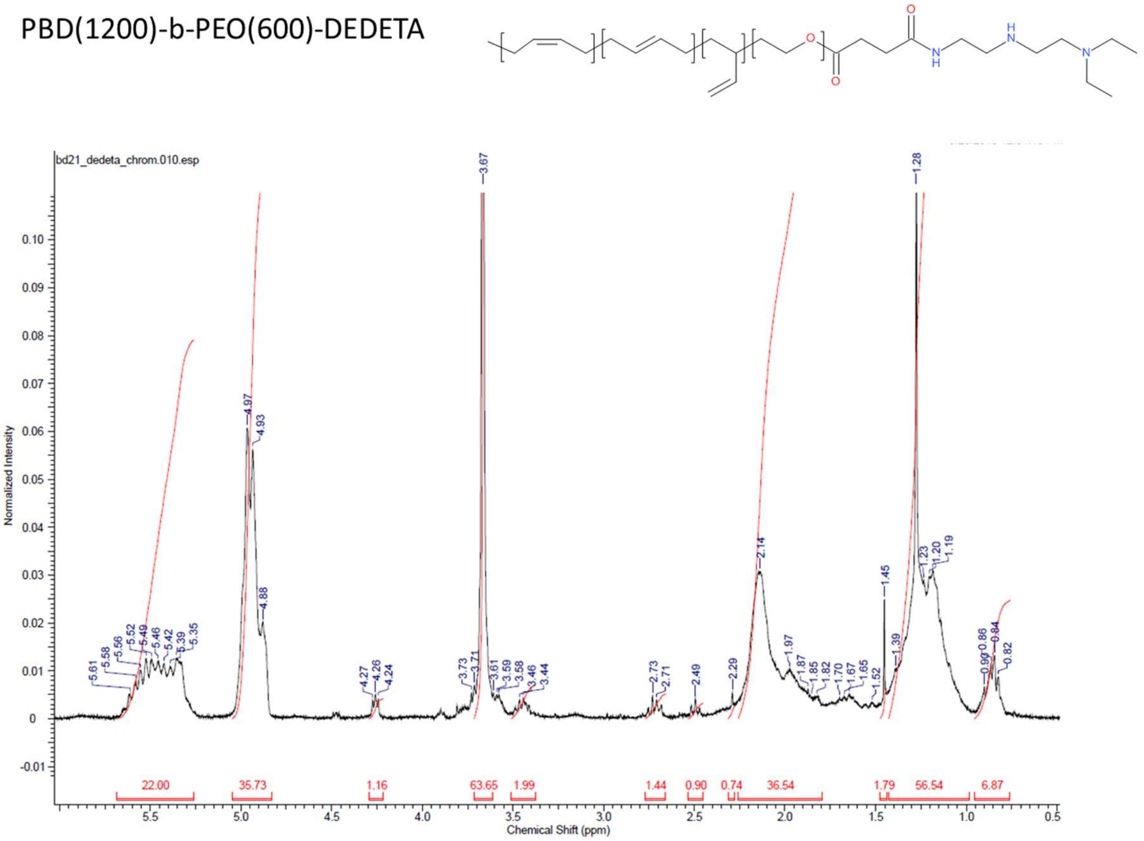

^{1}H-NMR ($CDCl_3$, 300 MHz, ppm): 8.21 (b, 0.7H, -CONH-), 5.45 (m, 24H, -C=CH- 1,2-BD, -HC=CH- c,t-1,4-BD), 4.94 (t, 40H, H_2C=C- 1,2-BD), 4.26 (t, 1.4H, -CH_2COO-), 3.66 (m, 60H, -CH_2OCH_2- EG), 3.2-3.1 (b, 0.7H, -NH-), 2.72 (q, 1.5H, -CH_2N-), 2.49 (t, 2.9H, -OOCCH_2CH_2COO-), 2.25-1.8 (m, 28H, -CH_2CH- 1,2-BD, -H_2C-C=C c,t-1,4-BD), 1.5-1.0 (m, 40H, -CH_2CH- 1,2-BD & alkyl-CH_2), 0.94-0.78 (m, 7H, alkyl-CH_3 & N(-termCH_3)$_2$)

PBD(1200)-b-PEO(600)-DEDETA

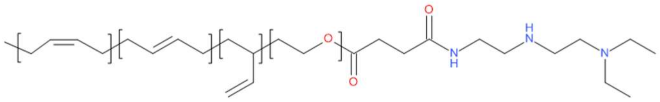

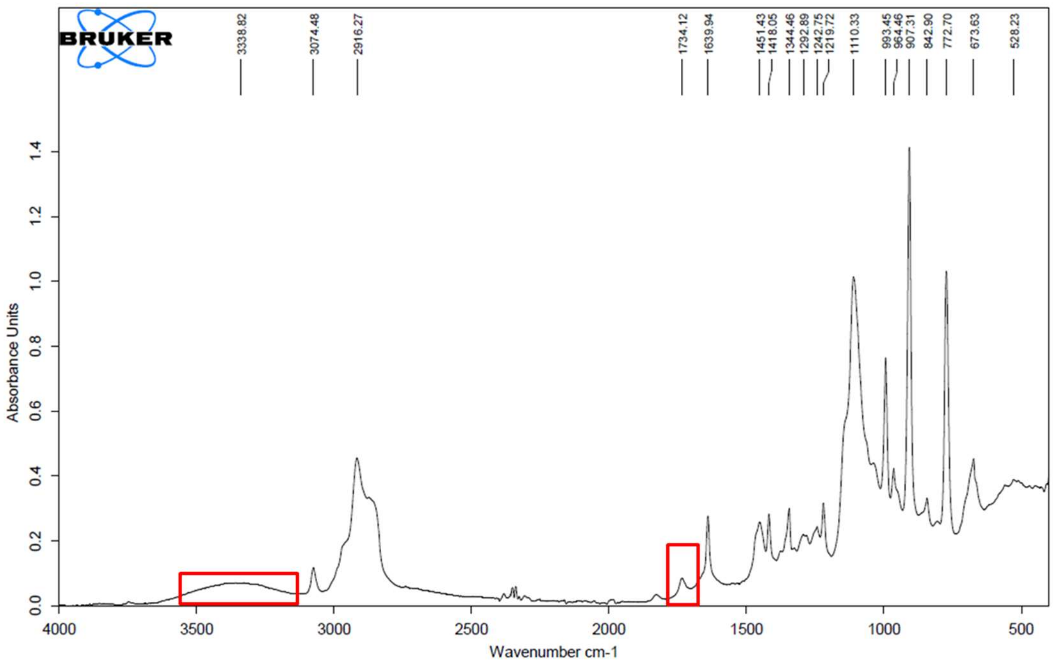

ATR-FTIR (powder, cm^{-1}): 3630-3150 (b, CONH, NH), 2972 (CH$_2$ BD), 2913 (CH$_2$ BD), 2890 (CH$_2$ EG), 1826 (comb.), 1735 (COOR), 1665 (CONH), 1640 (C=C BD), 1540 (CONH),), 1447 (CH$_2$ BD & EG), 1418 (CH$_2$ BD), 1378, 1349 (CH$_2$ EG), 1330 (COOR), 1300 (COOR), 1249 (CH$_2$ BD), 1101 (C-C & C-O EG), 1039 (C-C & C-O EG), 993 (=CH BD), 951 (CH$_2$ EG), 907 (=CH BD), 860 (CH$_2$ EG), 774, 675, 522

Characterization of magnetopolymersome assemblies

UVVIS and luminescence spectra of DEAC labeled-diblock copolymer assemblies

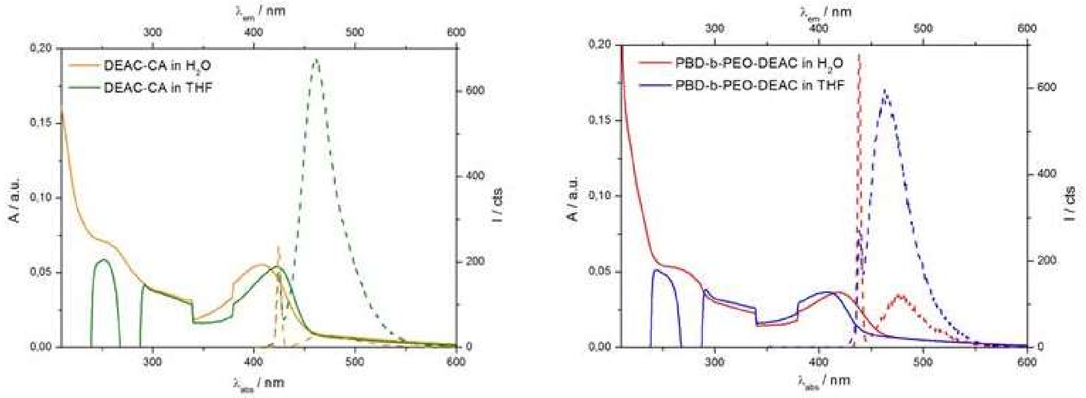

Fig S1. Normalized absorption and emission curves of DEAC-CA (left) and PBD(1200)-b-PEO(600)-DEAC (right) in various solvents.

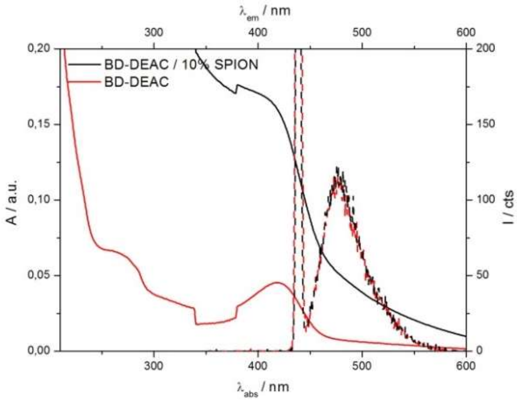

Fig S2. Normalized absorption and emission curves of PBD(1200)-b-PEO(600)-DEAC polymersomes with and without 10%w/w SPION loading.

Confocal images of cell-uptake for different preparation methods

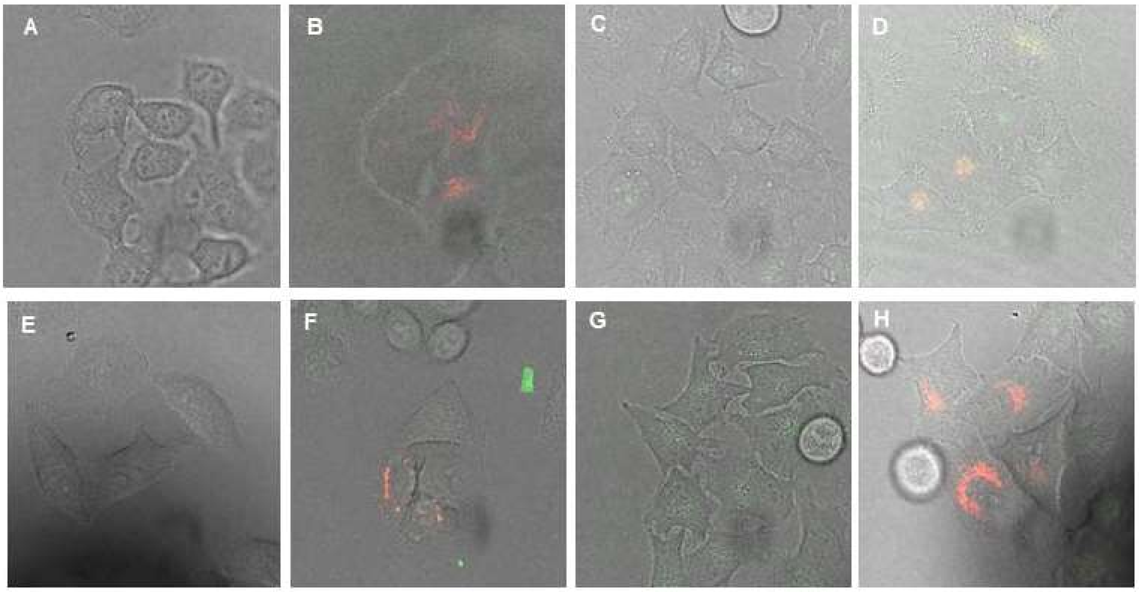

Fig S3: Confocal images of control magnetopolymersomes prepared via rehydration plus extrusion (100nm) at 2mg/ml. (A) HeLa cells (negative control), (B) HeLa cells expressing RFP after CellLight® staining, (C) HeLa cells after 12h incubation with cationic b-PEI adsorbed to the polymersomes (1% DEAC labeled), (D) co-localization of cationic fluorescent magnetopolymersomes with b-PEI adsorbed to the vesicle surface (green) in lysosomes (red) after 12h incubation, (E) HeLa cells after 12h incubation with DEDETA-modified polymersomes, (F) co-localization of (E), (G) HeLa cells after 12h incubation with EDOPC-blended PBD(1200)-b-PEO(600)-OH vesicles and (H) co-localization of (G).

Zeta potential distributions of various nanoparticle-diblock copolymer assemblies

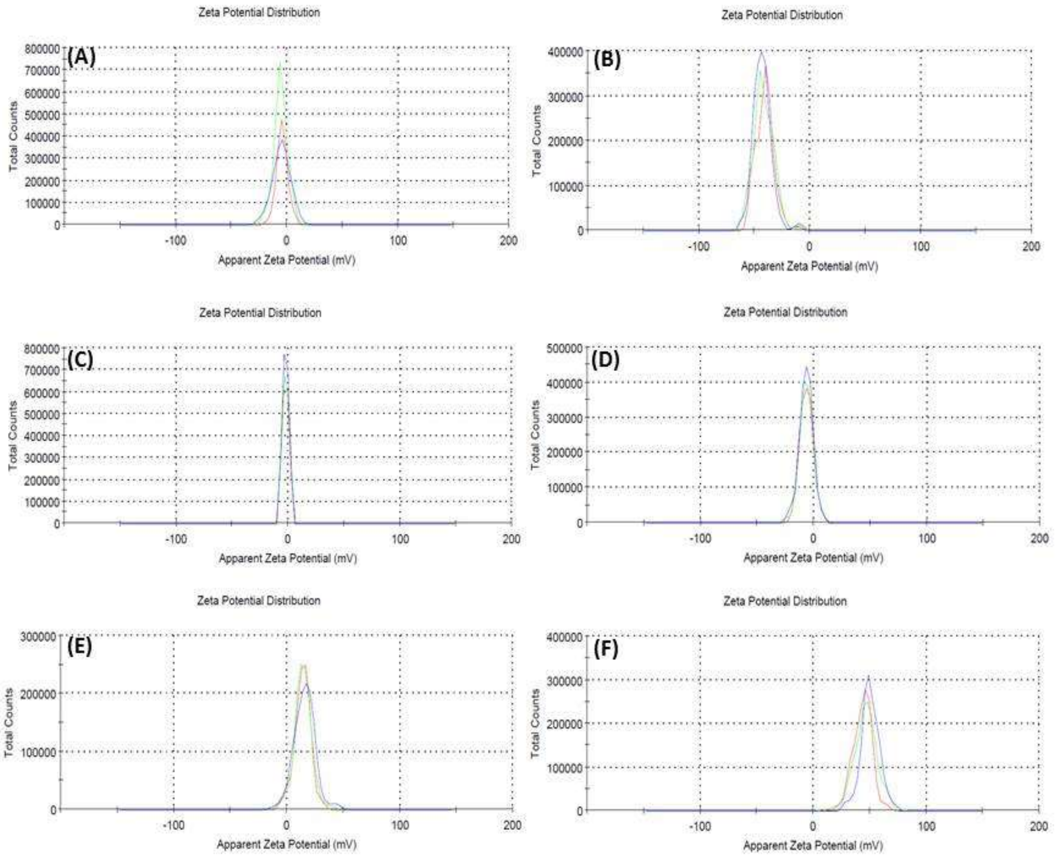

Fig S4: Zeta potential distributions of various magnetopolymersomes (10%w/w SPION) prepared at 5mg/ml in 1x PBS via rehydration and extrusion through 100nm polycarbonate membranes (Avestin). Samples were diluted 1:10 with Milli-Q water for measurements. (A) PBD(1200)-b-PEO(600)-OH (5% DEAC labeled), (B) PBD(1200)-b-PEO(600)-COOH, (C) PBD(1200)-b-PEO(600)-COOH / b-PEI(800) (10x; Sephadex G-75), (D) PBD(1200)-b-PEO(600)-DEDETA (50%n/n), (E) PBD(1200)-b-PEO(600) / EDOPC (30%n/n) and (F) POPC / EDOPC (30%n/n).

Table 1. Data chart for various magnetopolymersome preparations with d_H~140nm (prepared via rehydration plus extrusion)

sample	ζ-Pot [mV]
PBD-b-PEO-OH	-4.2 (±5.3)
PBD-b-PEO-COOH	-39.9 (±8.9)
PBD-b-PEO-COOH / b-PEI (10x; SEC)	-2.3 (±2.9)
PBD-b-PEO-DEDETA (50%)	-6.3 (±6.1)
PBD-b-PEO-OH / $DOPC^+$ (30%)	+15.6 (±9.7)
POPC / $DOPC^+$ (30%)	+45.3 (±8.5)

Gel-electrophoresis characterization of the supernatant before and after uptake

	pre-stained standard
	RPMI-1640
1	PBD(1200)-b-PEO(600)-DEAC<br>Fluorescent polymersomes
2	PBD(1200)-b-PEO(600)-DEAC /<br>30% SPION<br>Fluorescent magnetopolymeromses
3	PBD(1200)-b-PEO(600)-DEAC /<br>30% POPC<br>Fluorescent lipopolymersomes

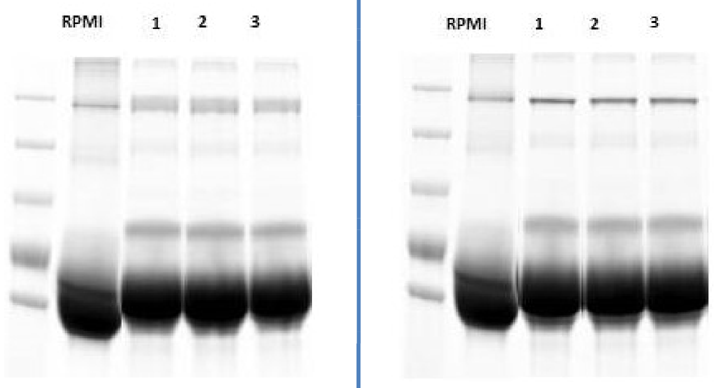

Fig S5: Gel electrophoresis characterization of the supernatants before and after incubation with magnetopolymerosmes.

Cytotoxicity chart for different preparation methods

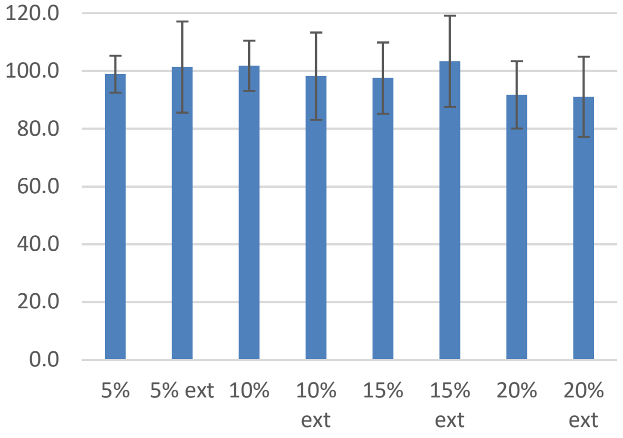

Fig S6: Cytotoxicity data of fluorescent magnetopolymersomes (1% DEAC, 10% SPION) prepared via solvent inversion at 2mg/ml and by rehydration plus extrusion (31x, 100nm polycarbonate membrane). Samples were incubated with HeLa cell lines for 12h before evaluated by Resazurin assays. Cell viability is evaluated as emission ratio 590/560.

Evaluation of iron content from uptake of cationic magnetopolymersomes

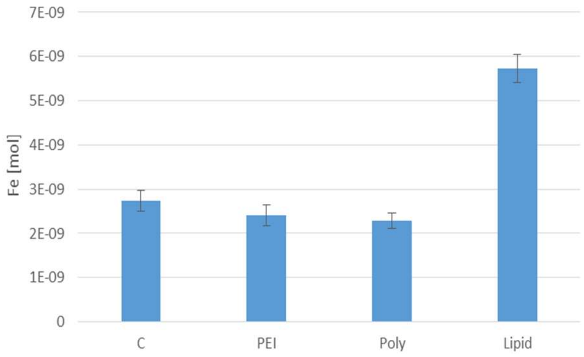

Fig S7: Data chart for iron uptake of cationic magnetopolymerosmes (10% SPION) as evaluated by Ferrozin assays (C-cellular background, PEI – b-PEI modified, Poly – DEDETA modified, Lipid – EDOPC modified PBD(1200)-b-PEO(600) polymersomes). Samples were prepared via solvent inversion at 2mg/ml and homogenized by post-extrusion (10x, 100nm).

References

(1) Hyeon, T.; Lee, S. S.; Park, J.; Chung, Y.; Na, H. B. Synthesis of Highly Crystalline and Monodisperse Maghemite Nanocrystallites without a Size-Selection Process. *J. Am. Chem. Soc.* **2001**, *123* (51), 12798–12801.

(2) Bixner, O.; Lassenberger, A.; Baurecht, D.; Reimhult, E. Complete Exchange of the Hydrophobic Dispersant Shell on Monodisperse Superparamagnetic Iron Oxide Nanoparticles. *Langmuir* **2015**, *31* (33), 9198–9204.

(3) Song, A.; Wang, X.; Lam, K. S. A Convenient Synthesis of Coumarin-3-Carboxylic Acids via Knoevenagel Condensation of Meldrum's Acid with Ortho-Hydroxyaryl Aldehydes or Ketones. *Tetrahedron Lett.* **2003**, *44* (9), 1755–1758.
